# Neuroanatomical normative modelling in frontotemporal lobar degeneration: higher heterogeneity in the behavioural variant

**DOI:** 10.1007/s00415-025-13378-5

**Published:** 2025-09-23

**Authors:** Srijan Konwar, Nikolett Hunyadvari, Annalena Venneri, James H. Cole, Martina Bocchetta

**Affiliations:** 1 Department of Psychology, College of Health, Medicine and Life Sciences, Centre for Cognitive and Clinical Neuroscience, Brunel University of London, London, UK; 2 Department of Neurodegenerative Disease, Dementia Research Centre, UCL Queen Square Institute of Neurology, University College London, London, UK; 3 Department of Computer Science, UCL Hawkes Institute, University College London, London, UK; 4 Department of Medicine and Surgery, University of Parma, Parma, Italy; 5 Department of Neuroscience, University of Sheffield, Sheffield, UK

**Keywords:** Frontotemporal lobar degeneration, Neuroanatomical normative modelling, Individual neuroimaging biomarkers, MRI

## Abstract

**Introduction:**

Frontotemporal lobar degeneration (FTLD) includes heterogenous diseases: behavioural variant frontotemporal dementia (bvFTD), primary progressive aphasias (PPA), progressive supranuclear palsy (PSP) and corticobasal syndrome (CBS). We applied neuroanatomical normative modelling to quantify individual atrophy patterns and heterogeneity within and between FTLD forms.

**Methods:**

We included 160 participants across FTLDNI and 4RTNI studies: controls (*n* = 15), bvFTD (*n* = 22), nfvPPA (*n* = 14), svPPA (*n* = 21), CBS (*n* = 43) and PSP (*n* = 45). Using cortical thickness and subcortical volumes from 3T MRIs, we applied normative modelling with a large healthy reference dataset (*n* = 58,836), further accounting for age, sex, and scanner. Outlier regions (*z* < – 1.96) were used to compute total outlier counts (tOC) and Hamming distances, capturing individual atrophy patterns and inter-subject dissimilarity.

**Results:**

bvFTD, svPPA, CBS and PSP showed significantly higher cortical tOC than controls, with all groups showing higher subcortical tOC than controls, especially svPPA and PSP. bvFTD, svPPA, CBS and PSP had significantly higher cortical Hamming distance scores than controls, with higher scores in bvFTD and svPPA than nfvPPA and PSP. svPPA and PSP had significantly higher subcortical scores than controls and CBS. Greater disease severity (measured using the Clinical Dementia Rating—CDR for PSP and CBS, and the CDR® plus NACC-FTLD global scores for FTD variants) was associated with increased tOC and dissimilarity, highlighting the link between clinical progression and neuroanatomical heterogeneity.

**Conclusions:**

The pronounced heterogeneity within and between FTLD subtypes (particularly in bvFTD) increases with disease progression and may reflect distinct underlying pathologies. This supports the development of subtype-specific biomarkers and emphasize the need for personalized diagnostic and therapeutic strategies.

**Supplementary Information:**

The online version contains supplementary material available at 10.1007/s00415-025-13378-5.

## Introduction

Frontotemporal lobar degeneration (FTLD) is an umbrella term that defines a set of heterogenous neurodegenerative diseases, with onset usually between 45 and 60 years of age [1]. Clinically, FTLD can manifest itself with different symptoms, such as cognitive and behavioural changes, language or motor difficulties, and can be diagnosed as behavioural variant frontotemporal dementia (bvFTD), primary progressive aphasias (PPA), progressive supranuclear palsy (PSP), or corticobasal syndrome (CBS). FTLD is also associated with different abnormal underlying pathology, such as tau, transactive response DNA binding protein 43 (TDP-43), or FET [2]. The evolution and types of symptoms and neuroanatomical signatures vary between and within FTLD-associated disorders, and there is no clear association between these signatures and the underlying pathology, so it is difficult to provide accurate diagnosis and prognosis to individual patients [3, 4]. Evidence from a network of memory clinics suggests that the FTD syndrome takes longer to diagnose and might be underdiagnosed compared with other forms of dementia (i.e., Alzheimer’s disease, AD), so it is paramount to develop measures that can aid precision medicine [5].

While case–control studies are ubiquitous in research, the overarching usage of group averages to describe a distinct clinical group can be problematic. This is because neurodegenerative diseases are not uniform due to a plethora of factors, from genetic to multiple pathologies and comorbidities that could result in the manifestation of idiosyncratic and heterogenous clinical phenotypes and disease progression [6]. For example, a study examined whether a group of patients with FTLD syndromes can be classified as an individual diagnostic entity or whether they shared common features placing them on a multidimensional spectrum. The study found that subject-specific scores overlapped across diagnostic groups and 62% met the criteria for more than one syndrome, with some groups having common overlapping syndromes: bvFTD with PSP or the semantic variant PPA (svPPA), non-fluent variant PPA (nfvPPA) with CBS or PSP [7]. Interestingly, this study also found that 44% of CBS patients had PSP-like features, while 30% of PSP patients had CBS-like features. This suggests that clinical subtypes are not mutually exclusive entities based on either clinical features or structural brain changes confined by a specific diagnostic boundary, rather these subtypes exist in a multidimensional spectrum [7]. Moreover, behavioural, linguistic and neuroanatomical heterogeneity was found across different PPA groups (svPPA, nfvPPA and logopenic variant PPA) [8] and even within the same clinical group [9].

Marquand and colleague introduced a normative modelling framework for neuroimaging to study deviations from a normative reference point of brain structure at the individual rather than the group level [10]. An advantage of this framework is that the normative distributions were defined based on a cohort of thousands of healthy participants [10].

To date, neuroanatomical normative modelling has been applied in developmental conditions such as autism spectrum disorder [11, 12], psychiatric conditions such as psychosis [13], schizophrenia [14] and obsessive–compulsive disorder [15]. More recently, this has been applied to different neurodegenerative diseases such as AD [16–18], dementia with Lewy bodies and Parkinson’s disease [19], but has yet to be applied to FTLD.

Neuroanatomical normative modelling allows the study of intragroup heterogeneity within each distinct clinical diagnostic group and paves the way for a novel approach to the assessment of neurological conditions alternative to examining between-group differences using averages [20]. It also becomes important to note that even within a single entity such as bvFTD, several subtypes (temporal-dominant, temporofrontoparietal, frontotemporal and fronto-dominant) have been reported in relation to volume loss. Volumetric loss in distinct regions has been observed in each subtype that performs differently in measures of cognitive tests of episodic memory, executive function and confrontation naming resulting in a heterogenous clinical phenotype [21]. Therefore, we aimed to apply this neuroanatomical normative model in a cohort of FTLD variants, specifically to examine the variation in individual spatial patterns of neuroanatomical heterogeneity in volume measures within the distinct FTLD subtypes, which include bvFTD, two language variants (svPPA and nfvPPA), CBS and PSP.

Firstly, we aimed to (1) assess intragroup neuroanatomical heterogeneity by measuring the outliers within each brain region for each clinical subtype to obtain variant-specific deviation patterns in brain regions (i.e., cortical thickness and subcortical volumes) as indicated by the total outlier count (tOC) and Hamming distance measures. Secondly, (2) assess intergroup differences in outlier patterns to investigate whether there are any significant differences in brain outlier regions that could be unique to each group. We hypothesised that the bvFTD group would reflect greater intra-group dissimilarity than the two language variant subtypes. While the two language variants would both show unique patterns specific to the temporal lobe that might result in overlapping regions, svPPA would show a more focal pattern in the left anterior temporal lobe with lower dissimilarity and a much more severe atrophy pattern than nfvPPA.

## Materials and methods

### Participants

Data were obtained from the Frontotemporal Lobar Degeneration Neuroimaging Initiative (FTLDNI) and the 4-Repeat Neuroimaging Initiative (4RTNI) database (http://4rtni-ftldni.ini.usc.edu/). FTLDNI and 4RTNI were launched in 2010 and early 2011 and funded through the National Institute of Aging and the Tau Research Consortium. More information is available at http://memory.ucsf.edu/research/studies/nifd and http://memory.ucsf.edu/research/studies/4rtni.

The FTLDNI and 4RTNI participants were recruited at four sites in North America: participants underwent a brain magnetic resonance imaging (MRI) scan and cognitive and clinical assessments such as the Mini-Mental State Examination (MMSE) and the Clinical Dementia Rating (CDR). For the FTLDNI patients, the global scores for the CDR® plus NACC-FTLD were also calculated, as a more accurate measure of disease severity for these FTD variants [22]. Exclusion criteria for patients included major psychiatric or neurologic comorbidity, sustained hypertension, and vascular diseases, while cognitively normal controls were included if they had a CDR score of 0 and an MMSE score ranging from 26 to 30, along with normal physical and mental health.

The project received ethical approval from Brunel University of London Research Committee (42307-NER-Mar/2023-44239-1).

The original sample consisted of 474 participants with volumetric T1-weighted MRIs, and sociodemographic, clinical, and cognitive data. After visual quality checks of T1-weighted MRIs images and their segmentations, 188 (39.7%) participants had to be excluded (6 CBS, 22 PSP, 55 bvFTD, 42 PPA, 63 healthy controls) due to significant motion artifacts or failed segmentation (e.g., often severe atrophy resulting in poor reconstruction of the cortical surface), and 69 (14.5%) due to unspecified or mixed diagnosis. The final sample included in the study consisted of 217 images, comprising 160 participants in the experimental sample: healthy controls (*n* = 15), bvFTD (*n* = 22), nfvPPA (*n* = 14), svPPA (*n* = 21), CBS (*n* = 43) and PSP Richardson’s syndrome (*n* = 45). Of the final sample of healthy individuals passing quality control (a total of 63 individuals), 57 healthy controls (21 males; age: 64.79 ± 7.99 years, range: 46–83 years) were included for model adaptation, as described below.

### MRI acquisition and processing

T1-weighted MRI data were collected using a three-dimensional magnetization-prepared rapid gradient-echo (MPRAGE) sequence on 3T Siemens Trio or GE MRI scanners with a slice thickness of 1 or 1.2 mm.

T1-weighted MRIs were processed using FreeSurfer version 6.0 recon‐all function (https://surfer.nmr.mgh.harvard.edu/) [23]. For each participant, cortical thickness and subcortical volumes were generated based on the Destrieux atlas [24]. This included mean cortical thickness and thickness values for 148 bilateral cortical regions, and volumes of 19 subcortical structures and total intracranial volume.

### Neuroanatomical normative modelling

Individual *z*-scores of cortical thickness and subcortical volume data adjusted for covariates were obtained using the PCN portal online platform (https://pcnportal.dccn.nl/) [25]. Specifically, we implemented a non-Gaussian Bayesian regression model, adjusting for unwanted noise from acquisition across multiple sites. The reference training set was a sample of 58,836 individuals from 82 different sites that was used to generate normative models for each brain region, with age, sex, and scanner type included as covariates. Further information about the specific parameters and models can be obtained from Rutherford et al. (2022) and Fraza et al. (2021) [26, 27].

We then calibrated these estimates to our specific dataset, using an adapted transfer learning approach [28]. The parameters of the reference normative model were recalibrated to our FTLDNI + 4RTNI datasets using 80% of randomly selected healthy controls and these were included in the adaptation set. The data from the remaining 20% controls (*n* = 15) and from the patients were included in the test data, and compared with these normative models, generating *z*-scores per region for each individual.

## Statistical analysis

### Demographic variables

Demographic variables were checked for normality using the Shapiro–Wilk test. A One-way Analysis of Variance (ANOVA) was used for variables that did not violate normality and Kruskall-Wallis for variables that did not pass normality checks. Chi-Square tests were used to analyse the relationships between categorical variables. All tests for demographic variables and normality checks were conducted using SPSS version 29 (SPSS Inc., Chicago, IL, USA). All other analyses were conducted using R version 4.4.1.

### Regional pairwise comparisons between diagnostic groups for cortical thickness and subcortical volumes

Between group comparisons were conducted on *z*-scores adjusted for age, sex and scanner type for each cortical thickness region (148 regions) and each subcortical volume region (19 bilateral regions which included the cerebellar cortex, thalamus, caudate, putamen, pallidum, hippocampus, amygdala, accumbens, ventral diencephalon and the brainstem) using the Kruskall-Wallis H due to violation of normality evidenced in checks conducted on the dependent variables stratified by clinical groups. We used Kruskall–Wallis test across all thickness and volumetric variables to account for unequal group size and heteroscedasticity, which could otherwise contribute to Type I errors. Post hocs were carried using Dunn’s test with further adjustments for multiple comparisons using false discovery rate (FDR). Between-group comparisons were conducted to examine group differences in mean cortical thickness (*z*-scores) and mean subcortical volumes (*z*-scores) using the Kruskall–Wallis H with post hocs using Dunn’s test with Bonferroni adjustment for multiple comparisons.

All between-group and pairwise comparisons were analysed using the Kruskal_test function in R. Only the FDR corrected *p*-values were mapped onto the brain regions using the Desterieux atlas for thickness and aseg atlas for subcortical volumes using the ggseg version 1.6.6 available in R [29]. All graphical visualizations of the post hocs were performed using the ggboxplot function from the ggplot2 package in R.

### Individualized brain markers: outliers and outlier distance scores

For each region, outliers were defined if the *z*-scores of the thickness or volumetric values were < − 1.96 (corresponding to the 2.5th percentile of the normative distribution). The total number of outliers (tOC) was calculated across the brain regions of each individual, and could range from 0 to 148 for the cortical thickness and from 0 to 19 for subcortical volumes.

After thresholding outliers into a binary variable (outlier = 1, non-outlier = 0), we calculated the pairwise Hamming distances between individuals. Hamming distance reflects the dissimilarity between two vectors of categorical datapoints [19], and were computed using the stringdist package available in R [30]. We used Hamming distances to compare pairs of participants within the same diagnostic group across regions. Between-group differences for median Hamming distances and tOC for thickness and volumes were examined using the Kruskall–Wallis H with post hocs using Dunn’s test with Bonferroni adjustment for multiple comparisons. In addition, the proportion of participants with outliers within each diagnostic group were calculated for each region for thickness and volumes and mapped on to the respective atlases. We also examined between group differences in outliers for each of the 148 cortical thickness regions and each subcortical volume region (19 regions) outliers using the Chi-Square function or Fisher’s Exact test function (chisq.test or fisher.test) adjusted for multiple comparisons using FDR in R.

### Analyses by disease severity within clinical groups

Additionally, all analyses listed above were repeated by stratifying the diagnostic groups using the CDR (for PSP and CBS groups) and the CDR® plus NACC-FTLD global scores (for bvFTD, svPPA, nfvPPA groups). Two categories within each diagnostic group were identified as a measure of disease severity, from very mild (global scores = 0.5) to clearly symptomatic (global scores ≥ 1). Intra-group pairwise comparisons were conducted using an independent samples t-test if both CDR/CDR® plus NACC-FTLD groups passed normality checks, Welch t-test for any violation of homogeneity of variance, and Mann–Whitney U test using normal approximation to account for ranked ties with continuity correction for any violation of normality. We excluded patients due to either missing CDR/CDR® plus NACC-FTLD global scores (2 CBS and 4 PSP/1 bvFTD and 1 nfvPPA) or having a CDR score of 0 (6 CBS and 4 PSP). However, we repeated the analyses combining these 10 CBS and PSP individuals with the CBS and PSP individuals with CDR = 0.5, respectively, labelling these two subgroups as “CDR ≤ 0.5”. Sample size for each severity group is reported in Supplementary Table 1. As the bvFTD subgroup with CDR® plus NACC-FTLD global scores = 0.5 includes only three patients, the results discussed below should be interpreted with caution.

## Results

### Demographic characteristics

The ANOVA showed significant differences in age between the diagnostic groups and controls included in the test analyses (Table 1). Post hocs analyses revealed that the PSP group was significantly older (68.78 ± 7.09 years) than controls (62.27 ± 6.24, *p* = 0.025), svPPA (62 ± 6.52, *p* = 0.004) and bvFTD (62 ± 5.57, *p* = 0.003). CDR and CDR® plus NACC-FTLD global scores were significantly different between diagnostic groups. In particular, controls showed significantly lower CDR global scores (median = 0.00) than svPPA (0.50, *p* = 0.002), PSP (0.50, *p* < 0.001), CBS (0.50, *p* < 0.001) and bvFTD (1.00, adjusted *p* < 0.001). nfvPPA showed significantly lower CDR global scores (median = 0.50) than bvFTD (1.00, *p* = 0.002). In addition, CDR® plus NACC-FTLD global scores revealed that the bvFTD (median = 1) had a higher mean rank of 34.90 compared with the nfvPPA (median = 1; mean rank = 20.12).

**Table 1:** Demographics and Clinical Characteristics of the testing cohort

	Controls (*N* = 15)	CBS (*N* = 43)	PSP (*N* = 45)	nfvPPA (*N* = 14)	svPPA (*N* = 21)	bvFTD (*N* = 22)	Statistics
Age (Mean) ± SD^a^ Range	62.27 ± 6.24 49–70	66.23 ± 7.04 53–82	68.78 ± 7.09 55–85	66.07 ± 8.11 54–81	62 ± 6.52 50–74	62 ± 5.57 47–74	*F*(5,154) = 5.110, *p* < 0.001
CDR global score (Median and IQR)^b^ Range	0 (0)	0.5 (0.5) 0–3	0.5 (0.5) 0–2	0.5 (0) 0–0.5	0.5 (0.3) 0.5–1	1 (1.5) 0.5–2	*χ*^2^(5) = 40.947, *p* < 0.001
CDR® plus NACC-FTLD global score (Median and IQR)^b^ Range	–	–	–	1 (0.5) 0.5–1	1 (0.5) 0.5–2	1 (1.0) 0.5–2	*χ*^2^(2) = 9.098, *p* = 0.011
Sex (male/female)^c^	6/9	21/22	19/26	7/7	12/9	15/7	*χ*^2^ = 5.050, *p* = 0.410

^a^One-way Analysis of Variance (ANOVA)

^b^Kruskall-Wallis

^c^Chi-Square

### Group differences in cortical thickness and subcortical volumes (*z*-scores)

All patient groups showed average negative *z*-scores for cortical thickness, with the svPPA (median = − 1.775, interquartile range [IQR] = 1.89) and bvFTD (median = − 1.473, IQR = 2.875) groups having the lowest mean cortical thickness (Supplementary Fig. 1A). There were significant differences in cortical thickness between the groups, *χ*^2^ (5, *N* = 160) = 29.382, *p* < 0.001, with a large effect size (*η*^2^ = 0.158). Both bvFTD and svPPA (*p* < 0.001) had significantly lower cortical thickness than controls (median = 0.165, IQR = 0.995) and PSP (median = − 0.560, IQR = 1.305, *p* < 0.025 and *p* < 0.044, respectively) (Supplementary Fig. 1A).

When looking at the neuroanatomical regional patterns, these were consistent with the literature (Supplementary Fig. 1B): compared with controls, bvFTD showed reduced thickness mainly in the frontal cortex, svPPA mainly in the anterior temporal cortex, and nfvPPA mainly in the inferior frontal, fronto-opercular and anterior insular regions. When compared with controls, CBS showed significantly lower cortical thickness in the left superior precentral and central sulcus, bilateral precentral gyrus, right postcentral sulcus, and PSP in the right middle-anterior cingulate gyrus and sulcus bilaterally, left precentral gyrus and inferior frontal sulcus.

All patient groups also showed an average negative *z*-scores for the subcortical volumes, with svPPA (median = − 1.309, IQR = 0.863) and PSP (median = − 1.062, IQR = 1.166) being the groups with the lowest values (Supplementary Fig. 2A). There were significant differences in the mean subcortical volumes between the groups, *χ*^2^ (5, *N* = 160) = 15.150, *p* = 0.010, with a moderate effect size (*η*^2^ = 0.066), with only PSP showing significantly smaller *z*-scores than controls (median = − 0.351, IQR = 0.746, *p* = 0.006).

When looking at the regional patterns, as expected bvFTD showed smaller bilateral accumbens, bilateral hippocampus and left putamen volumes than controls, svPPA showed significantly smaller volumes than controls in the amygdala and hippocampus bilaterally and in the left accumbens. Compared with controls, CBS showed smaller volumes in the right putamen and thalamus, while PSP showed smaller volumes in the thalamus, ventral diencephalon, and brainstem (Supplementary Fig. 2B).

### Pairwise comparisons by disease severity within clinical groups for differences in mean cortical thickness and subcortical volumes (*z*-scores)

The bvFTD (CDR® plus NACC-FTLD global scores ≥ 1) group (mean = − 2.384, SD = 1.738) had significantly lower mean cortical thickness than the bvFTD (CDR® plus NACC-FTLD global scores = 0.5) group (mean = 0.068, SD = 0.513, *t*(11.855) = 4.850, *p* < 0.01) with a large effect size (Cohen’s d of 1.914, 95% CI [0.808, 2.979]). This difference was localised mainly in the left superior occipital gyrus, inferior insula, and superior frontal sulcus, and in the right posterior-ventral cingulate gyrus and inferior frontal gyrus (Supplementary Fig. 3A).

Similarly, there was a statistically significant difference between PSP subgroups, *t*(18.797) = 2.284, *p* = 0.034, indicating that the most severe PSP group (CDR global scores ≥ 1) (mean = − 1.209, SD = 1.526) had significantly lower mean cortical thickness than the PSP group with CDR global scores = 0.5 (mean = − 0.236, SD = 0.762) with a large affect size (Cohen’s d of 0.807, 95% CI [0.059, 1.536]). The difference was mainly in the right central sulcus, superior temporal gyrus and lateral orbital sulcus, in the left parahippocampal gyrus and middle frontal sulcus (Supplementary Fig. 3A). When considering CDR ≤ 0.5, the pattern of differences was similar to when excluding those with CDR = 0 in both PSP and CBS subgroups (Supplementary Fig. 3A-1). However, given the larger sample size for CBS, there was now a statistically significant difference between CBS subgroups, *t*(24.029) = 2.096, *p* = 0.047, with that the most severe CBS group (CDR global scores ≥ 1) (mean = − 1.621, SD = 2.099) showing lower mean cortical thickness than the CDR global scores ≤ 0.5 (mean = − 0.427, SD = 1.253) with a medium effect size (Cohen’s d of 0.691, 95% CI [0.010, 1.359]). This difference was mainly localised in the left posterior ventral cingulate and parahippocampal gyrus, calcarine and inferior frontal sulcus (Supplementary Fig. 3A-1).

The bvFTD (CDR® plus NACC-FTLD global scores ≥ 1) group (mean = − 1.137, SD = 1.107) had significantly lower subcortical volumes than the bvFTD (CDR® plus NACC-FTLD global scores = 0.5) group (mean = 0.371, SD = 0.703, *t*(19) = 2.255, *p* = 0.036) with a large effect size (Cohen’s d of 1.406, 95% CI [0.089, 2.690]). This difference was localised in the left accumbens, and caudate and putamen bilaterally (Supplementary Fig. 3B). No statistically significant difference was detected for the other groups in the mean subcortical volumes, with few individual regions resulting significant in PSP and CBS (Supplementary Fig. 3B).

### Group differences in tOCs for cortical thickness and subcortical volumes

**Fig. 1:**
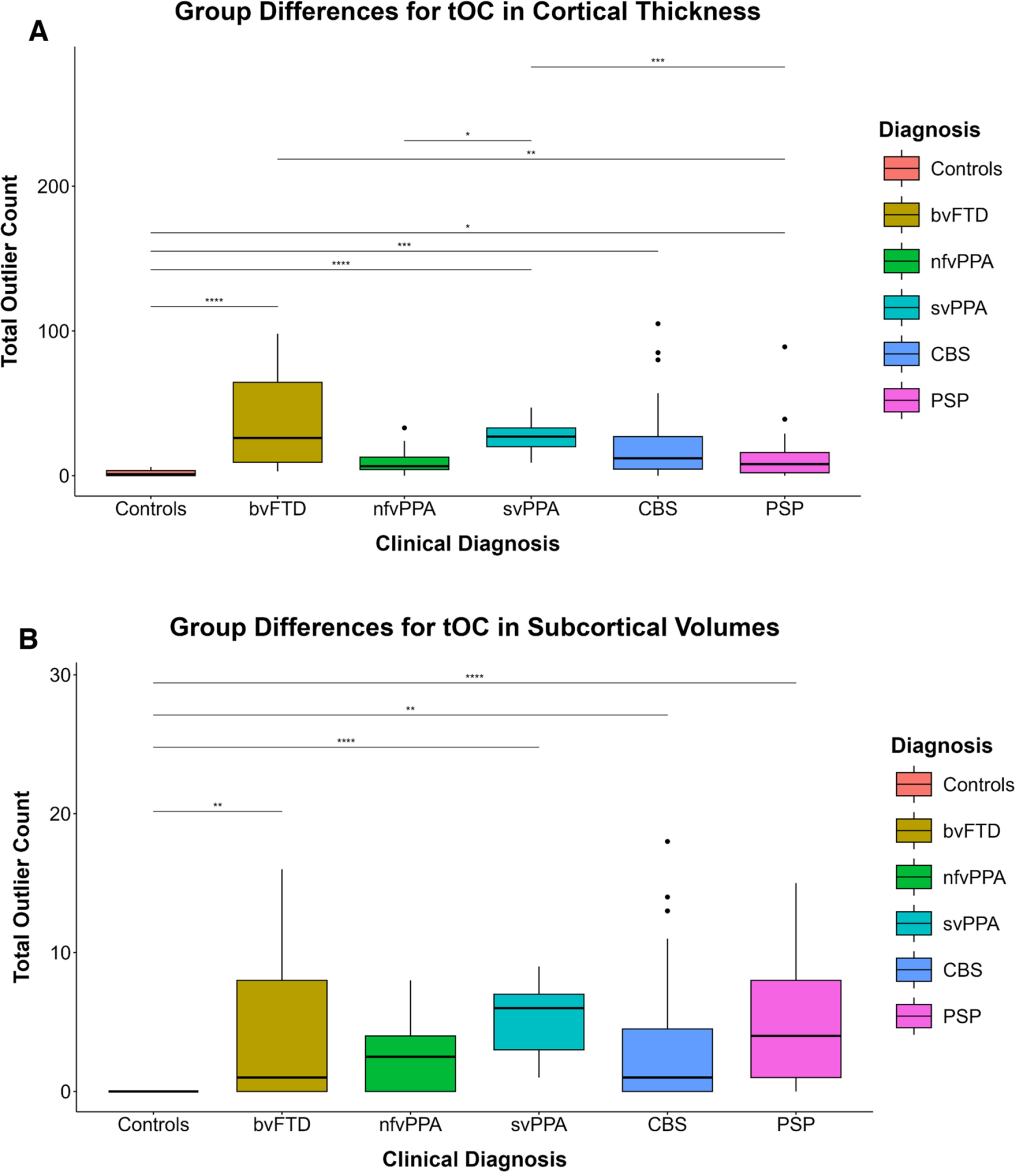
**A.** Post hoc pairwise comparisons show significant between group differences in total Outlier Count (tOC) for cortical thickness (148 regions) after Bonferroni adjustment. Statistically significant *p*-values were denoted by the following: ^*^p < 0.05, ^**^p < 0.01, ^***^p < 0.001, ^****^p < 0.0001. **B.** Post hoc pairwise comparisons show significant between group differences in total Outlier Count (tOC) for subcortical volumes (19 regions) after Bonferroni adjustment. Statistically significant *p*-values were denoted by the following: ^*^p < 0.05, ^**^p < 0.01, ^***^p < 0.001, ^****^p < 0.0001

There were significant differences between the groups, *χ*^2^ (5, *N* = 160) = 54.14, *p* < 0.001 in tOC for cortical thickness regions with a large effect size (*η*^2^ = 0.319). bvFTD (median = 26, IQR = 55.25,* p* < 0.001), svPPA (median = 27, IQR = 13,* p* < 0.001), CBS (median = 12, IQR = 22.5,* p* < 0.001) and PSP (median = 8, IQR = 14,* p* = 0.033) had significantly higher tOC than controls (median = 1, IQR = 3.5). The bvFTD (median = 26, IQR = 55.25,* p* = 0.002) had significantly higher tOC than PSP (median = 8, IQR = 14). Both nfvPPA (median = 6.5, IQR = 8.5, *p* = 0.013) and PSP (median = 8, IQR = 14, *p* < 0.001) had significantly lower tOCs than svPPA (median = 27, IQR = 13) (Fig. 1A).

There were significant differences between the groups, *χ*^2^ (5, *N* = 160) = 34.102, *p* < 0.001 in tOC for subcortical volume regions with a large effect size (*η*^2^ = 0.189). bvFTD (median = 1, IQR = 8, *p* = 0.004), svPPA (median = 6, IQR = 4, *p* < 0.001), CBS (median = 1, IQR = 4.50, *p* = 0.009) and PSP (median = 4, IQR = 7, *p* < 0.001) had significantly higher tOC than controls (median = 0, IQR = 0) (Fig. 1B).

### Pairwise comparisons by disease severity within clinical groups for differences in tOC for cortical thickness and subcortical volumes

In bvFTD, the severe group (CDR® plus NACC-FTLD global scores ≥ 1) had significantly higher tOC (median = 34, IQR = 55.75; U = 4, *p* = 0.024, Mann–Whitney U test) than the mild group (median = 7, IQR = 2) with a rank biserial correlation of -0.852 indicating that the effect size was large. Similarly, in PSP, the severe group had significantly higher tOC for cortical thickness (median = 17, IQR = 17.50; *U* = 72.50, *p* = 0.004, Mann–Whitney *U*) than the milder group (median = 5.50, IQR = 11.00) with a rank biserial correlation of -0.561 indicating that the effect size was large (Supplementary Fig. 4A). The results when considering the PSP CDR ≤ 0.5 group were similar (data not shown).

The severe bvFTD subgroup showed statistically significant higher tOC for subcortical volumes (median = 1.50, IQR = 7.00; *U* = 6 *p* = 0.034, Mann–Whitney *U* test) than the CDR® plus NACC-FTLD global scores = 0.5 group (median = 0.00, IQR = 0.00), with a rank biserial correlation of -0.778 indicating that the effect size was large (Supplementary Fig. 4B). No statistically significant difference was detected for the other groups and brain regions.

### Group differences in median Hamming distance for cortical thickness and subcortical volumes

**Fig. 2:**
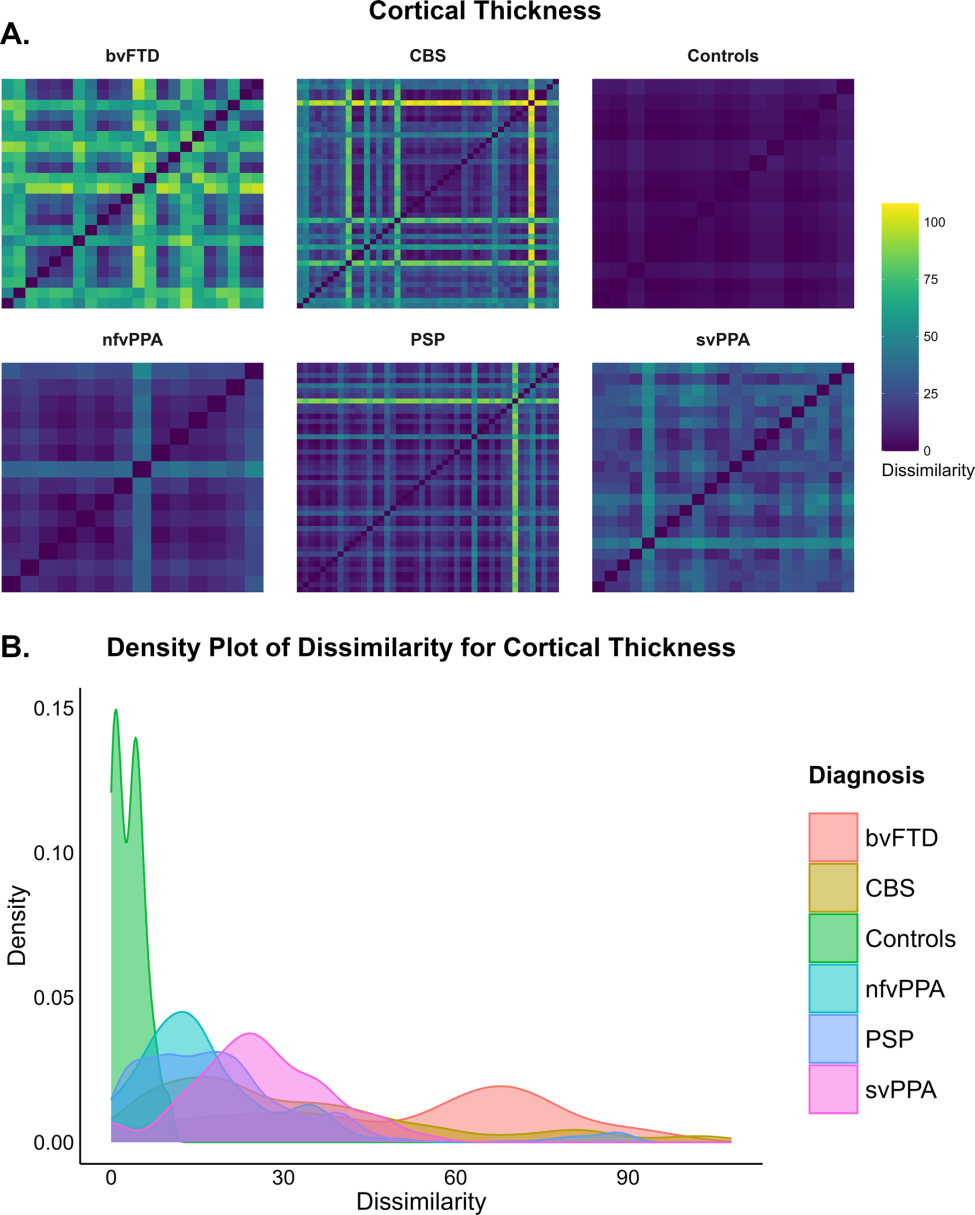

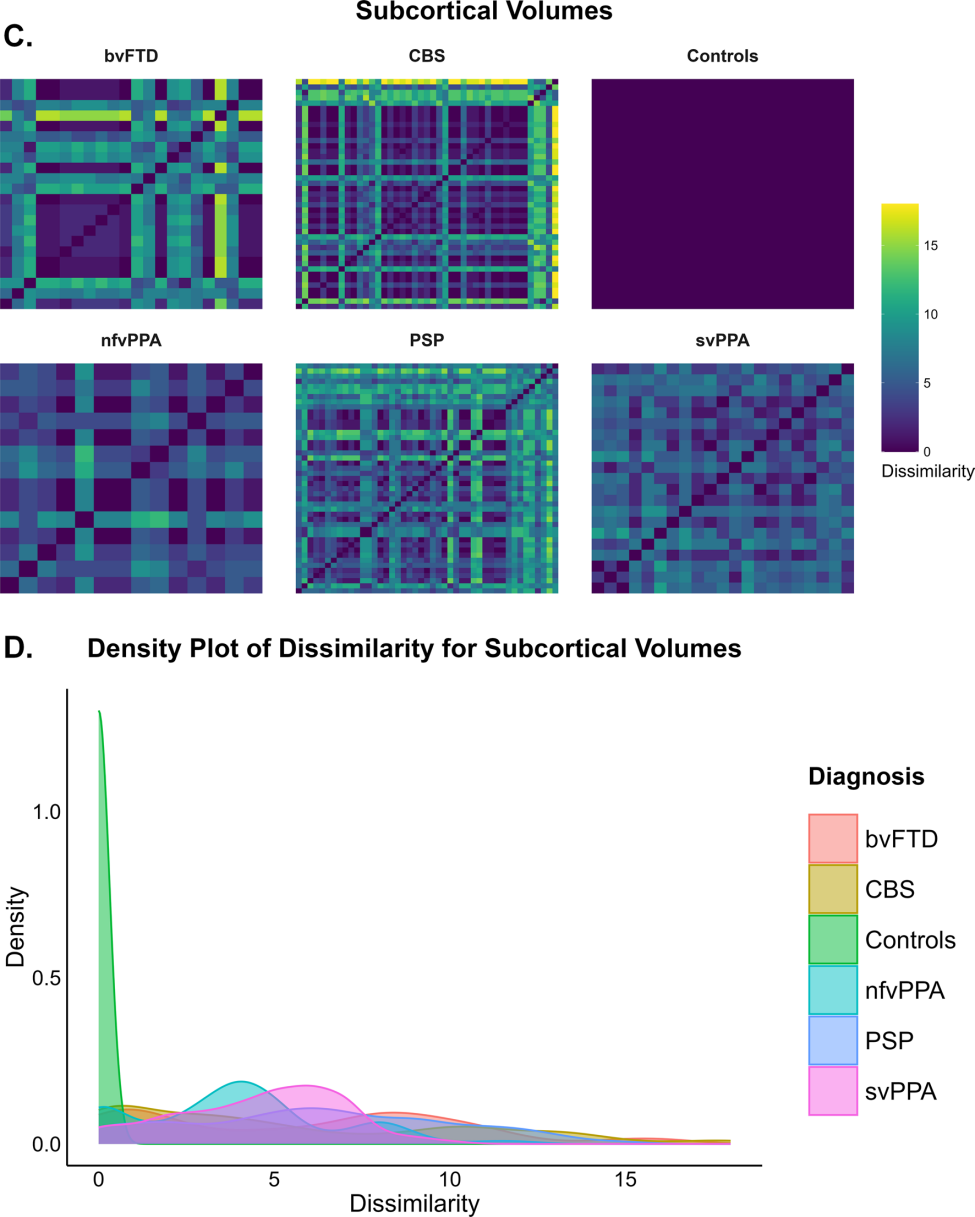
**A** depicts the outlier Hamming distance heatmaps for cortical thickness for each of the diagnostic groups. The x and y axes indicate the participants within each diagnosis. The darker colours indicate lower Hamming distance or less dissimilarity between participants across brain regions within each group (i.e. Controls have Hamming distance of 0 and is reflected by dark blue) and the brighter colours such as yellow indicate higher Hamming distance or greater dissimilarity between participants across brain regions within each group. **B** depicts the outlier Hamming distance density plots that highlights the spread of outlier dissimilarity across each of the diagnostic groups. **C** depicts the outlier Hamming distance heatmaps for subcortical volumes for each of the diagnostic groups. **D** depicts the outlier Hamming distance density plots that highlights the spread of outlier dissimilarity across each of the diagnostic groups

There were significant differences between the groups, *χ*^2^ (5, *N* = 160) = 89.76, *p* < 0.001, in median Hamming distance for cortical thickness regions with a large effect size (*η*^2^ = 0.550). Figure 2A and B show heatmaps and density plots indicating that within-group dissimilarity for thickness was highest in bvFTD. Pairwise comparisons showed that bvFTD (median = 36.5, IQR = 34, *p* < 0.001), svPPA (median = 24, IQR = 8.5, *p* < 0.001), CBS (median = 17, IQR = 15.25, *p* < 0.001) and PSP (median = 13, IQR = 10, *p* = 0.001) had significantly higher median Hamming distance for cortical thickness than controls (median = 2, IQR = 3.5). bvFTD has significantly higher Hamming distance than nfvPPA (median = 12, IQR = 5.5, *p* < 0.001), CBS (*p* = 0.002) and PSP (*p* < 0.001). svPPA has significantly higher Hamming distance than nfvPPA (*p* = 0.01) and PSP (*p* = 0.003) (Supplementary Fig. 5A).

There were significant differences between the groups, *χ*^2^ (5, *N* = 160) = 61.19, *p* < 0.001 in Hamming distance for subcortical volume regions with a large effect size (*η*^2^ = 0.365), with PSP being the most dissimilar group, followed by svPPA. Figure 2C and D show heatmaps and density plots indicating that within-group dissimilarity for volumes was highest in the PSP. bvFTD (median = 2, IQR = 7, *p* < 0.001), nfvPPA (median = 3.5, IQR = 1.5, *p* = 0.001), svPPA (median = 5, IQR = 2, *p* < 0.001), CBS (median = 2, IQR = 3.5 *p* < 0.001) and PSP (median = 5, IQR = 3, *p* < 0.001) showed significantly higher median Hamming distance for subcortical volumes than controls (median = 0, IQR = 0). Only PSP (median = 5, IQR = 3, *p* = 0.002) showed significantly higher median Hamming distance than CBS (median = 2, IQR = 3.5) (Supplementary Fig. 5B).

### Pairwise comparisons by disease severity within clinical groups for differences in median Hamming distance for cortical thickness and subcortical volumes

The PSP subgroup with CDR ≥ 1 showed significantly higher median Hamming distance for cortical thickness (median = 21.50, IQR = 14.75) than the PSP with CDR = 0.5 (median = 12.50, IQR = 7.50; *U* = 76 *p* = 0.006), with a rank biserial correlation of -0.539 indicating that the effect size was large (Supplementary Fig. 6A). Similar results were found when considering the PSP CDR ≤ 0.5. The CBS subgroup with CDR ≥ 1 showed significantly higher median Hamming distance for cortical thickness (median = 21.00, IQR = 31.00) than the CBS with CDR ≤ 0.5 (median = 16.50, IQR = 11.00; *U* = 124 *p* = 0.035), with a rank biserial correlation of -0.392 indicating a medium effect size (Supplementary Fig. 6A). Moreover, the most severe bvFTD subgroup had significantly higher median Hamming distance for subcortical volumes (median = 2.50, IQR = 6.00) than the mild bvFTD subgroup (median = 1.00, IQR = 0.00; U = 6 *p* = 0.034, Mann–Whitney U test), with a rank biserial correlation of -0.778 indicating that the effect size was large (Supplementary Fig. 6B). Supplementary Fig. 7A shows heatmaps indicating that within-group dissimilarity was highest for thickness in the bvFTD and PSP clinically severe groups. Overall, the CDR/CDR® plus NACC-FTLD global scores = 0.5 subgroups have lower Hamming distance values in cortical thickness than the more severe subgroups. For subcortical volumes (Supplementary Fig. 7B), the level of dissimilarity is almost comparable between clinical subgroups, except within bvFTD. Both PSP and CBS subgroups with CDR ≤ 0.5 showed similar heterogeneity than their CDR = 0 respective subgroups (Supplementary Fig. 7C).

### Regional pairwise comparisons of outliers in cortical thickness

**Fig. 3:**
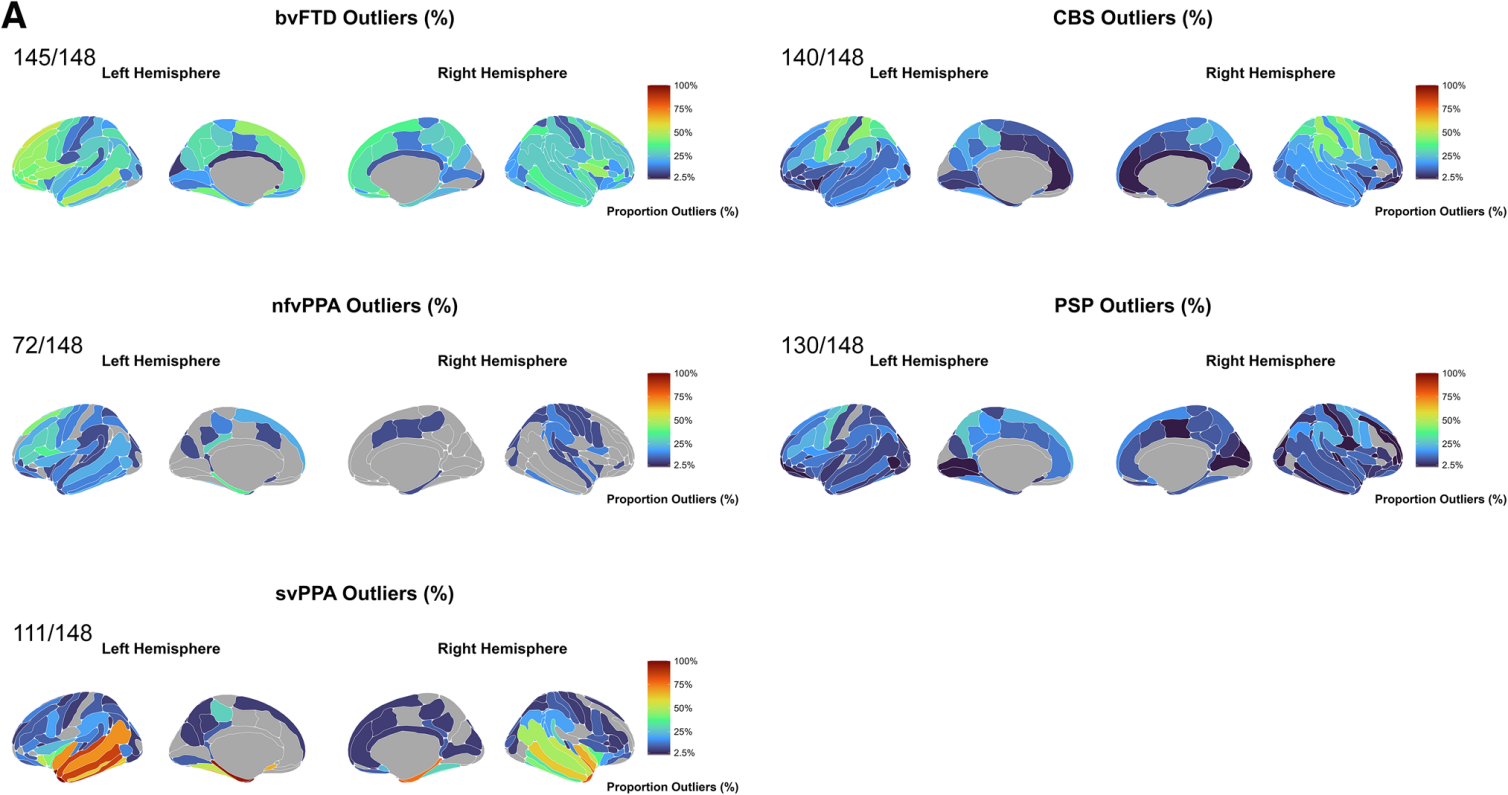

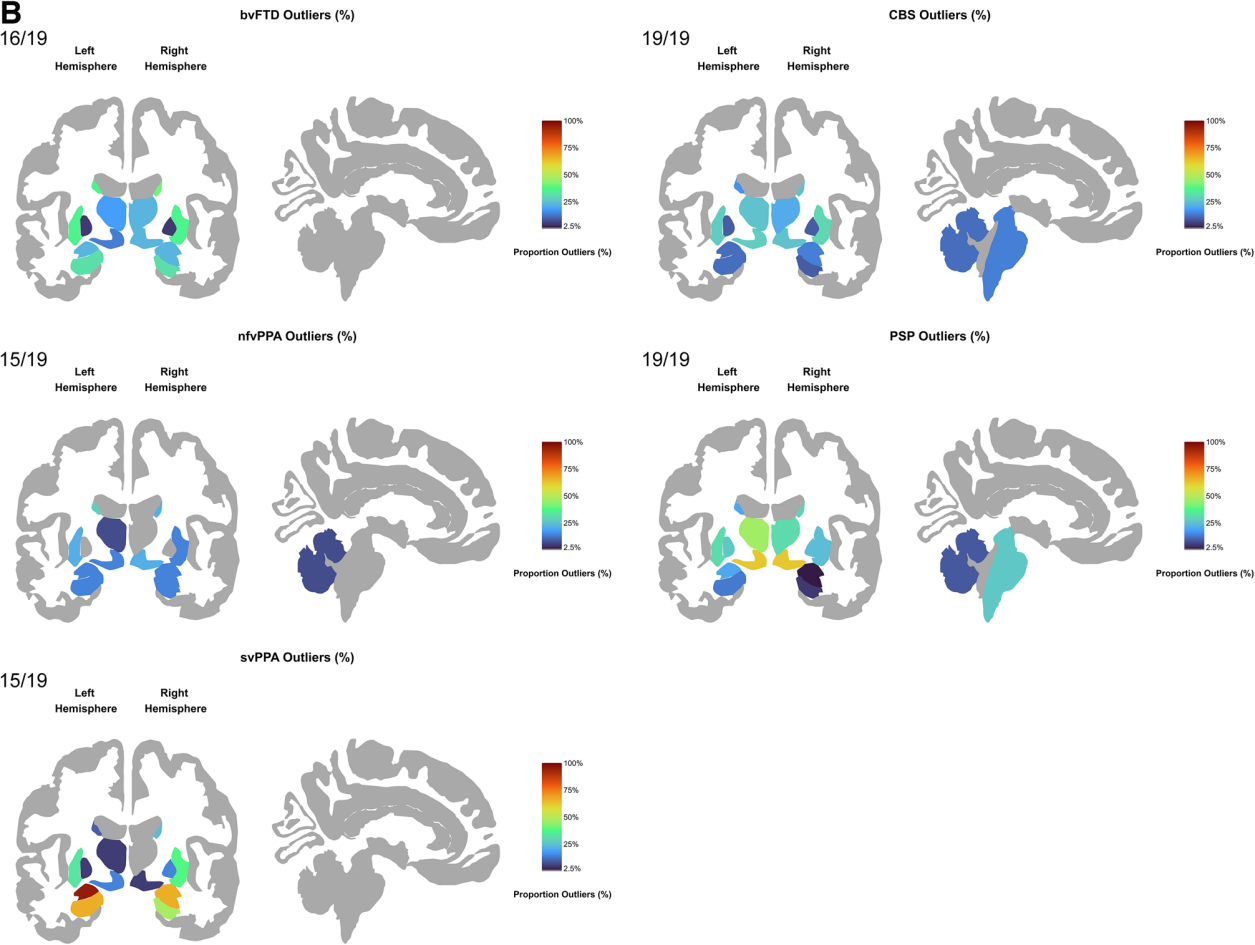
**A**. Percentage of patients with outliers within each diagnostic group have been mapped at each of the 148 regions. The colour bar reflects the percentage of outliers from 0% (darker colours such as dark blue) to 100% (bright colours such as dark orange). The grey colour represents regions where the proportion of outliers is between 0 and < 2.5%. **B**. Percentage of patients with outliers within each diagnostic group have been mapped at each of the 19 regions. The colour bar reflects the percentage of outliers from 0% (darker colours such as dark blue) to 100% (bright colours such as dark orange). The grey colour represents regions where the proportion of outliers is between 0 and < 2.5%. *Note*: The nucleus accumbens is not depicted in the images.

Overall, as visible in Fig. 3A, bvFTD patients had outliers in 145 cortical regions, with over 50% of patients having outliers predominantly in the left hemisphere regions (e.g., inferior, middle and superior frontal gyrus, and middle temporal gyrus). svPPA patients had outliers in 111 regions, with 70% of patients having these outliers in the temporal lobes bilaterally (temporal poles as well as in the superior, inferior and middle temporal). Interestingly, ≥ 85% of svPPA patients had outliers localised in the left temporal lobe, with all patients (100%) having outliers in the left temporal pole and left planum polare of the superior temporal gyrus. nfvPPA patients had outliers in 72 cortical regions, with a higher proportion of patients (> 30%) with outliers in the left superior and inferior frontal gyrus and parahippocampal gyrus. CBS patients had outliers in 126 regions with > 40% of patients showing outliers in the bilateral precentral and postcentral gyrus, left superior precentral sulcus, and right supramarginal gyrus. PSP patients had outliers in 130 regions, with ≥ 22% patients having outliers in the bilateral precentral gyrus and superior precentral sulcus, left precuneus, superior and inferior frontal gyrus, inferior precentral sulcus, and right supramarginal gyrus.

When compared with the other patient groups (svPPA, CBS and PSP), bvFTD showed significantly higher outlier count in the left middle frontal sulcus, left inferior frontal gyrus and left antero-lateral sulcus. svPPA showed significantly higher outlier count than bvFTD, nfvPPA, CBS and PSP in the temporal pole, parahippocampal gyrus and superior temporal gyrus bilaterally. CBS showed significantly higher outlier count than PSP and svPPA in the left superior parietal lobule, bilateral postcentral sulcus, right central sulcus and right intraparietal sulcus and transverse parietal sulci (Supplementary Figs. 8A, 8B, 8C).

### Regional pairwise comparisons by disease severity within clinical groups for outliers in cortical thickness

Overall, the bvFTD (CDR® plus NACC-FTLD global scores ≥ 1) group had outliers in 145 cortical regions, with ≥ 60% of patients having outliers predominantly in the left hemisphere (e.g., inferior frontal gyrus, superior and middle frontal sulcus, Supplementary Fig. 9A). The bvFTD (CDR® plus NACC-FTLD global scores = 0.5) group had outliers in 16 regions with ≥ 33% of patients having outliers predominantly in the left hemisphere, particularly in the left lateral orbital sulcus (67% of patients).

svPPA (CDR® plus NACC-FTLD global scores ≥ 1) group had outliers in 98 cortical regions with ≥ 93% of patients having outliers predominantly in the left hemisphere (e.g., lateral superior temporal gyrus, inferior and middle temporal gyrus) with the parahippocampal gyrus, planum polare of the superior temporal gyrus and temporal pole having all patients (100%) with outliers. The svPPA CDR® plus NACC-FTLD global scores = 0.5 group had outliers in 63 regions with ≥ 83% patients having outliers predominantly in the left parahippocampal gyrus, and all patients (100%) with outliers in the left planum polare of the superior temporal gyrus and temporal pole.

nfvPPA (CDR® plus NACC-FTLD global scores ≥ 1) group had outliers in 60 cortical regions with ≥ 42% patients having outliers predominantly in the left hemisphere (e.g., posterior-dorsal cingulate gyrus, lateral occipito-temporal gyrus, parahippocampal gyrus, inferior temporal gyrus and inferior frontal sulcus). nfvPPA (CDR® plus NACC-FTLD global scores = 0.5) group had outliers in 35 regions with ≥ 33% of patients having outliers predominantly in the left hemisphere, specifically in the inferior frontal gyrus (50% of patients) (Supplementary Fig. 9A).

There were no significant differences in outliers for any pairwise comparisons between disease severity subgroups for cortical thickness after FDR adjustments (Supplementary Figs. 9A and 9B).

### Regional pairwise comparisons of outliers in subcortical volumes

Overall, as shown in Fig. 3B, bvFTD had outliers in 16 subcortical regions with 30% of patients showing outliers in the bilateral accumbens nucleus, caudate, hippocampus and putamen. svPPA had outliers in 15 subcortical regions, with ≥ 40% of patients showing outliers in the left accumbens, bilateral amygdala and hippocampus. Interestingly, 95% of svPPA patients had outliers in the left amygdala. nfvPPA had outliers in 15 subcortical regions with ≥ 20% of patients showing outliers in the left accumbens and putamen, bilateral caudate, and right ventral diencephalon.

CBS had outliers in 19 subcortical regions, with ≥ 20% of patients having outliers in the left accumbens, right caudate, bilateral putamen, thalamus, and ventral diencephalon. PSP also had outliers in 19 regions, with ≥ 20% of patients showing outliers in the accumbens, caudate, pallidum, putamen, thalamus and ventral diencephalon bilaterally, and in the left amygdala. In addition, both the PSP (27%) and CBS (14%) groups had patients with outliers in the brainstem, while none of the three other FTD variants had any outliers in this region (Fig. 3B).

Compared with controls, bvFTD showed significantly higher outlier count in the caudate, putamen and left accumbens, while svPPA in the bilateral amygdala, hippocampus and left accumbens, and PSP in the bilateral ventral diencephalon, thalamus and left putamen (Supplementary Fig. 10).

The bvFTD group showed significantly higher outlier count than PSP in the right hippocampus and amygdala. The PSP group showed significantly higher outlier count than bvFTD, svPPA, nfvPPA and CBS primarily in the bilateral ventral diencephalon. The svPPA group showed significantly higher outlier count than bvFTD, nfvPPA, CBS and PSP, primarily in the bilateral amygdala (Supplementary Figs. 11A, 11B, 11C).

### Regional pairwise comparisons by disease severity within clinical groups for outliers in subcortical volumes

Overall, bvFTD (CDR® plus NACC-FTLD global scores ≥ 1) group had outliers in 16 subcortical regions with ≥ 44% patients having outliers predominantly in the bilateral caudate (Supplementary Fig. 12A). The left accumbens showed outliers for 50% of patients. Surprisingly, none of the mildly affected bvFTD patients showed outliers in any of the subcortical regions.

svPPA (CDR® plus NACC-FTLD global scores ≥ 1) group had outliers in 14 subcortical regions with ≥ 80% of patients showing outliers predominantly in the left hippocampus and right amygdala. All patients (100%) in this group showed outliers in the left amygdala. svPPA (CDR® plus NACC-FTLD global scores = 0.5) group had outliers in 12 regions with ≥ 66% of patients having outliers predominantly in the left accumbens, with the left amygdala having the highest proportion of patients (83%) with outliers.

nfvPPA (CDR® plus NACC-FTLD global scores ≥ 1) group had outliers in 14 subcortical regions with ≥ 28% of patients having outliers predominantly in the left accumbens (43%) and in the left caudate and amygdala, bilateral ventral diencephalon. nfvPPA (CDR® plus NACC-FTLD global scores = 0.5) group had outliers in 10 regions with ≥ 16% of patients having outliers predominantly in the bilateral caudate, putamen, hippocampus, accumbens, right amygdala and ventral diencephalon (Supplementary Fig. 12A).

There were no significant differences in outliers for any pairwise comparisons between disease severity subgroups for subcortical volumes after FDR adjustments (Supplementary Figs. 12A and 12B).

## Discussion

In this study, we utilised neuroanatomical normative modelling to identify individual atypical patterns in cortical thickness and subcortical volumes across FTLD subtypes. Overall, these results suggest that each FTLD-specific variant shows both between-group differences as well as within-group heterogeneity, which is reflected by the spatial distribution and numerosity of outliers. The results were in line with our expected hypothesis that the bvFTD group would show the highest intra-group heterogeneity. The Hamming distance for cortical thickness indicated that within-group dissimilarity was the greatest in the bvFTD as compared with all the diagnostic groups, for the numbers of regions with abnormal values compared with the normative population, for the variety of regions involved, and for the proportion of patients showing outliers. In terms of number of outliers in cortical regions, the bvFTD were followed by svPPA, CBS, PSP, and nfvPPA. For number of outliers in subcortical regions, svPPA was the group with the highest number of outliers followed by PSP, nfvPPA, CBS and bvFTD.

When looking at Hamming distance for both cortical and subcortical regions, bvFTD, PSP and CBS were the groups with the highest dissimilarity, with svPPA the most homogeneous group.

bvFTD outliers were more widespread with most regions having a moderately higher proportion of patients with outliers, particularly in the medial prefrontal, dorsolateral and temporal regions, and sparing the posterior regions. This is in line with the literature on similar alterations in both volume and metabolism, and reflects the behavioural presentation of these patients [21, 31–34].

While for bvFTD, the high dissimilarity scores were the result of the combined effect of various widespread outliers, in svPPA the outliers were much more focal to the anterior temporal lobe, hippocampus and amygdala, particularly in the left hemisphere, showing the highest proportion of patients with outliers out of all the diagnostic groups. The pattern on the right hemisphere mirrored the one that was seen on the left, with a lower proportion of outliers. These results were in accordance with the observed neuroimaging signatures [35], demonstrating that the left antero-temporal pole and medio-temporal structures are severely affected in the early stages of svPPA, with the right side following a similar sequential pattern of neuroanatomical involvement [36, 37]. This is a well-known finding in clinical practice, as svPPA patients are seen by clinicians when atrophy has already become apparent [38]. svPPA patients tend to show very localised atrophy, similar clinical presentation and progression of symptoms (breakdown of semantic memory, anomia and impaired single word comprehension). The relative homogeneity in this group compared with the other FTLD can be related to the common underlying pathology seen in these forms, nearly always TDP-43 type C [2].

The nfvPPA group showed fewer outliers than other groups and a lower overall intra-group heterogeneity. The outliers were in the regions typically affected in this form: left inferior frontal cortex and superior frontal cortex, with atrophy in these regions being associated with fluency difficulties [32, 39, 40]. The relative less extreme deviation from the norm in nfvPPA can be partially explained by the presence of a less severe disease for this subgroup of patients, as indicated by lower global scores on the CDR® plus NACC-FTLD.

Similarly to svPPA, CBS also showed outliers in localised regions, specifically in the precentral, postcentral cortex, the superior temporal gyrus and the middle-posterior cingulate. This finding is in accordance with another study that found smaller cortical thickness in the CBS in similar regions [41].

We found that the CBS group had moderate intra-group heterogeneity in the cortical thickness, which could be discussed in light of potentially different underlying pathologies and subtypes, as shown by a recent study [42]. Scotton and colleagues have demonstrated the presence of different subtypes in CBS, one with a clear subcortical involvement and mainly associated with 4-repeat tauopathies, and one with cortical atrophy in the frontal, parietal and occipital cortex, more frequently associated with Alzheimer’s pathology.

PSP patients exhibited outliers in cortical and subcortical regions overlapping with those of CBS. However, the proportion of PSP patients with outliers in the cortical regions was smaller compared with CBS. The PSP group showed a higher number of outliers in the subcortical volumes, particularly in the brainstem, bilateral thalamus and ventral diencephalon, in line with previous work [41, 43]. The cohort of PSP patients included in this study are PSP with Richardson’s syndrome, and as reported by Scotton et al. (2023) [44], this group tends to show more subcortical atrophy than cortical.

As shown by studies in other conditions, the within-group dissimilarity tends to be the highest in the most severe clinical groups, whether it is AD as compared with MCI [16, 17], or whether it is dementia with Lewy bodies as compared with Parkinson’s disease [19]. Here, we separated the patients into less and more severely affected using the global scores of the CDR (for PSP and CBS) or of the CDR® plus NACC-FTLD global score for bvFTD and PPA forms, as this scale includes modules for language and behaviours, which are more appropriate to capture disease severity in these FTD variants. We demonstrated that there is a difference in atrophy stratified by disease severity within each diagnostic group, particularly evident in bvFTD for both thickness and subcortical volumes. The most severe subgroup had significantly lower mean thickness and volumes, higher tOC (thickness and volumes) and higher median Hamming distance (volumes) than the bvFTD with CDR® plus NACC-FTLD global score = 0.5. The results for bvFTD are supported by another study that found that while bvFTD patients with a CDR global score of 0.5 had atrophy restricted to the anterior frontal lobe, paralimbic, limbic and subcortical regions, patients with a CDR global score of 1.0 and above showed more extensive frontal atrophy as well as bilaterally and involving more posterior regions [45]. The severely affected bvFTD subgroups were also much more dissimilar in the subcortical volumes, when compared with the bvFTD with CDR® plus NACC-FTLD global score = 0.5, who showed barely any dissimilarity. This was followed by the svPPA and this was highlighted by the presence of variant-specific outliers, tOC and variant-specific dissimilarity patterns based on thickness and volumetric data. This pattern of more heterogeneity and more outliers in the most severe stages of the disease was also statistically significant for the PSP subgroups, and shows the same trend in CBS, nfvPPA and svPPA forms. Including individuals with a CDR global score of 0 among the milder forms of PSP and CBS resulted in similar patterns to those observed when considering only individuals with a CDR global score of 0.5.

The results presented in this study highlight some ways that normative models can be utilised to characterise heterogeneity of brain structural alterations at the individual level in a cohort that included distinct FTD subtypes as well as cases of CBS and PSP Richardson’s syndrome. It also allowed us to explore the degree of spatial overlap by using individual *z*-scores to classify extreme deviations and to measure this deviation from a reference trajectory without confining us to a standard group template or average. Aside from the classical variant-specific pattern we observed in the FTLD forms, we mapped the extreme deviations at the individual level, which provided us with a robust estimate of variant-specific intra-group heterogeneity. Finally, we have also demonstrated that normative modelling is useful in characterising the heterogeneity within clinical forms with different degree of disease severity.

## Limitations

Due to the cross-sectional nature of the study, we could not account for individual progression and this design limits inference about clinical progression. We compared clinical forms by grouping them by their disease severity, but this still does not account for individual differences. Moreover, we used CDR scales to classify patients by disease severity, but these tools have been originally developed for other neurodegenerative conditions and may not fully capture the diverse symptoms of FTLD-associated diseases. Future studies should look at specific correlations between cognition, biomarkers and neuroanatomical heterogeneity in FTLD patients, including multimodal imaging metrics that can better indicate the complexity of brain alternations, by capturing additional brain change, beside cortical thickness and volume differences. Moreover, it is crucial to investigate the longitudinal progression of the individual from their neuroanatomical signature baseline. It will be important to factor in different rates of change that affect disease trajectory, and how useful the individual maps will be for predicting progression in FTLD [20]. Verdi et al. (2023) suggested that tOC can be a good prognostic neuroimaging indicator in AD [16, 18], but we are yet to demonstrate whether tOC could be useful to stratify patients with any form of dementia for clinical purposes. Another limitation is that we applied a conventional outlier threshold for *z*-scores (< − 1.96) based on the normative distribution, as per previous work [16–19]. At present, there is no definitive or optimal threshold to define an outlier for a specific disease and opting for a 2.5% percentile might have been too conservative considering our small sample size. Moreover, our findings are based on the subcortical and cortical regions as defined by the Destrieux atlas: these region-specific delineations may influence the observed distribution of anatomical involvement across groups.

Although we found significant differences in heterogeneity between disease severity groups for measures such as tOC and median Hamming distance, these results need to be taken with caution due to the relatively small and not equally distributed sample size and for the cut-off point of CDR global scores of 0.5 and ≥ 1. The limited sample size (including within diagnostic groups) limits the statistical power for robust between-group comparisons and it was partly driven by the strict exclusion criteria, as we prioritized high-quality scans and inputs to ensure methodological robustness. We recognize that this may limit the generalizability of our findings and reduce the representativeness of the cohort, particularly due to the likely exclusion of individuals with more advanced disease and severe atrophy. Future work is needed to explore the inclusion of lower-quality MRI scans and segmentations, and broader clinical variability to enhance the applicability of the approach across diverse patient populations and in clinical settings.

Moreover, we do not have access to the specific underlying pathology of each individual, which was not available in the cohort and it is difficult to determine in vivo. Future studies integrating *post* *mortem* data on the specific proteinopathies (i.e., tau, TDP-43, or FET) for each individual patient are essential to better interpret the observed neuroanatomical heterogeneity. Although the cohort is presumed to be sporadic, we do not have genetic confirmation that all participants tested negative for known FTLD-associated mutations, which could provide additional information on neuroanatomical patterns and heterogeneity. Furthermore, the lack of biomarker evidence cannot rule out possible diagnostic errors. Our findings are exploratory in nature, however, and future studies with larger, biomarker-confirmed cohorts will be essential to validate and extend these observations.

## Conclusion

Normative modelling allowed us to capture subtle differences in neuroanatomical heterogeneity across FTLD subtypes using two structural measures (cortical thickness and subcortical volumes). While all subgroups exhibited some degree of heterogeneity, the extent varied markedly, with bvFTD in particular, but also PSP and CBS, showing more pronounced variability than other forms. This highlights important subtype-specific patterns that may reflect underlying pathological differences. By further exploring summary metrics such as tOC from normative modelling in conjunction with clinical, cognitive, genetic and neuropathological data in larger cohorts, we can provide evidence to improve clinical prognostic accuracy using a patient-centric approach at the individual level and reduce diagnostic ambiguity.

## Supplementary Information

Below is the link to the electronic supplementary material.

Supplementary file 1 (DOCX 36194 KB)

## Data Availability

Data used in preparation of this article were obtained from the Frontotemporal Lobar Degeneration Neuroimaging Initiative (FTLDNI) and the 4-Repeat Tauopathy Neuroimaging Initiative (4RTNI) databases (https://4rtni-ftldni.ini.usc.edu/ and https://ida.loni.usc.edu/login.jsp). The investigators at FTLDNI and 4RTNI contributed to the design and implementation of FTLDNI and 4RTNI and/or provided data, but did not participate in analysis or writing of this report.
