# Supplementary material for: Neuroanatomical normative modelling in frontotemporal lobar degeneration: higher heterogeneity in the behavioural variant
Source: J Neurol. 2025 Sep 23;272(10):642. doi: 10.1007/s00415-025-13378-5 (PMC12457516; doi:10.1007/s00415-025-13378-5)
Supplement: Supplementary file 1 — (DOCX 36194 KB) [file 415_2025_13378_MOESM1_ESM.docx]

**
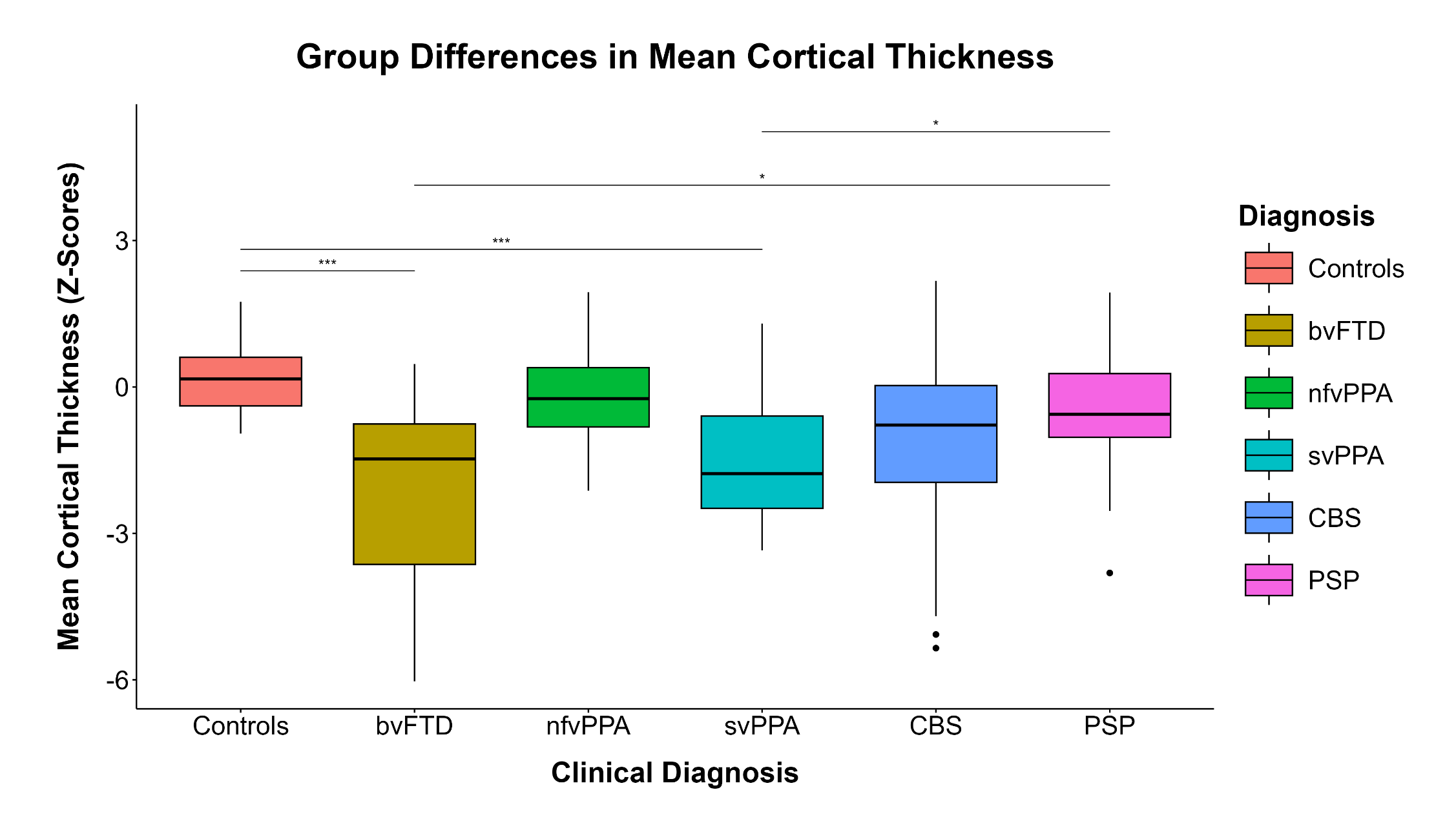
**

**Supplementary Figure 1A.** *Post hoc* pairwise comparisons show significant between group differences in mean cortical thickness after Bonferroni adjustment. Statistically significant p-values were denoted by the following: ^*^*p* < 0.05, ^***^*p* < 0.001.

**
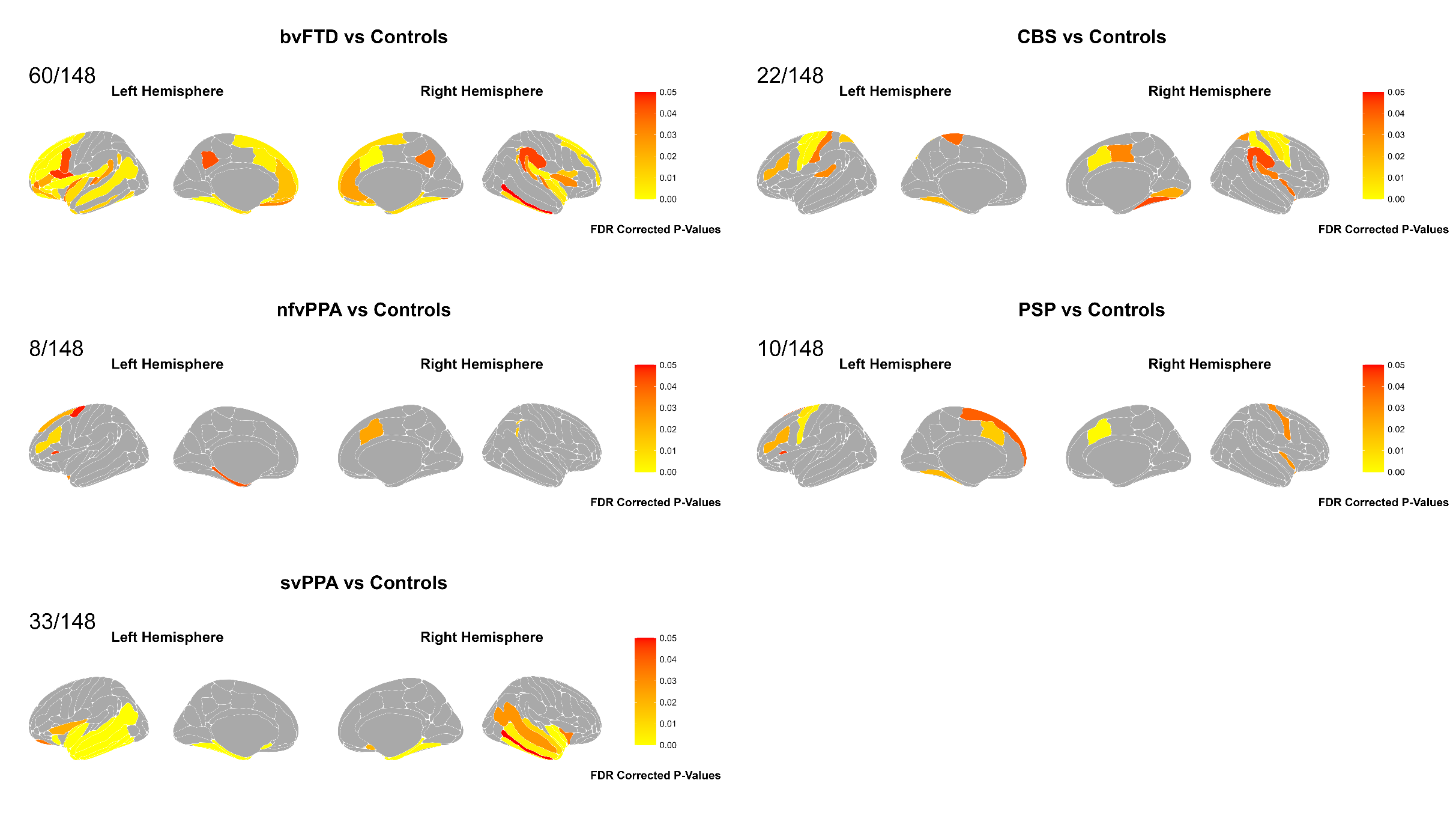
**

**Supplementary Figure 1B.** FDR adjusted p-value maps show significant between group differences for cortical thickness in different diagnostic groups vs Controls at each region.

**
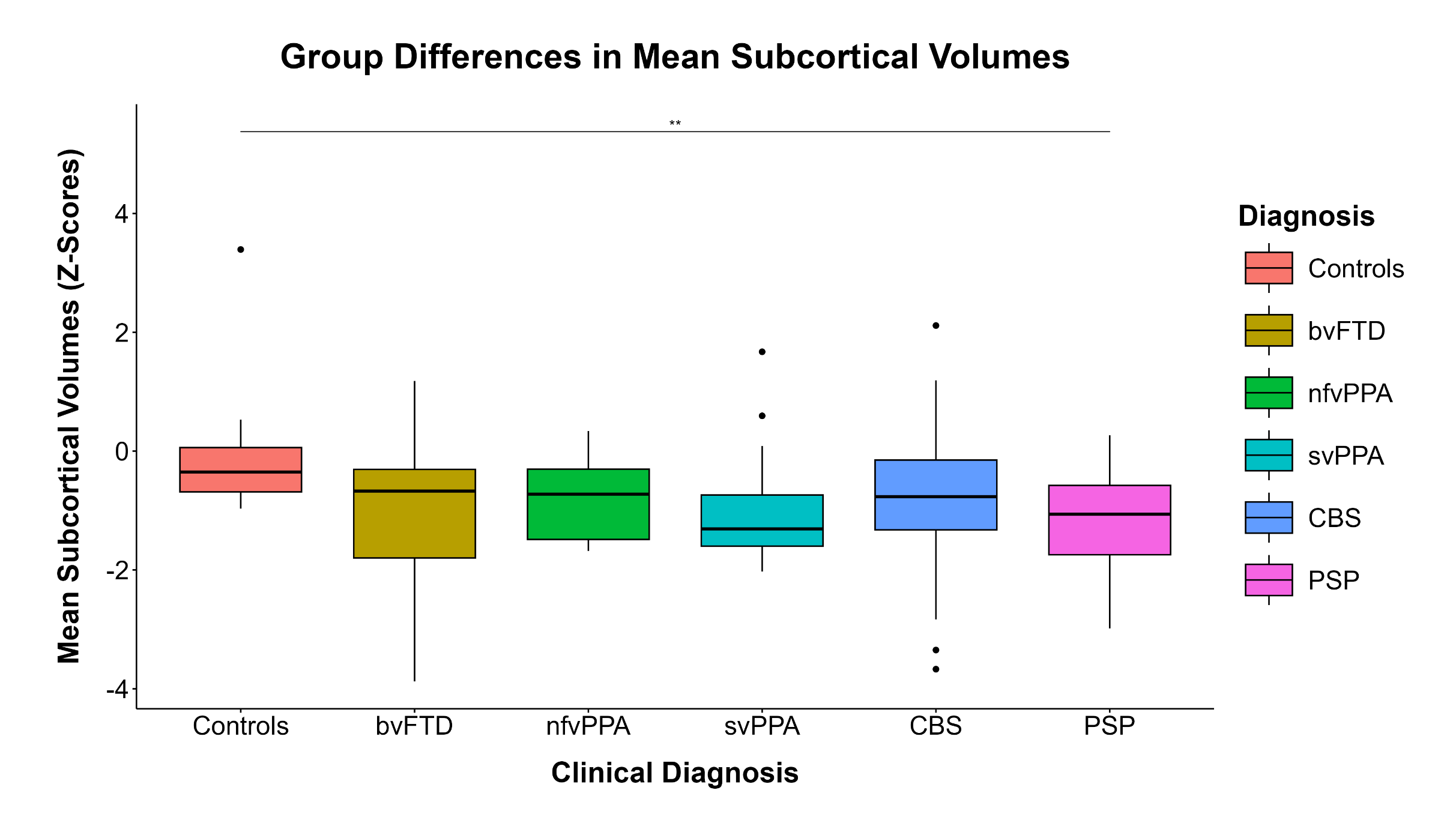
**

**Supplementary Figure 2A.** *Post hoc* pairwise comparisons show significant between group differences in mean subcortical volumes after Bonferroni adjustment. Statistically significant p-values were denoted by the following: ^**^*p* < 0.01.


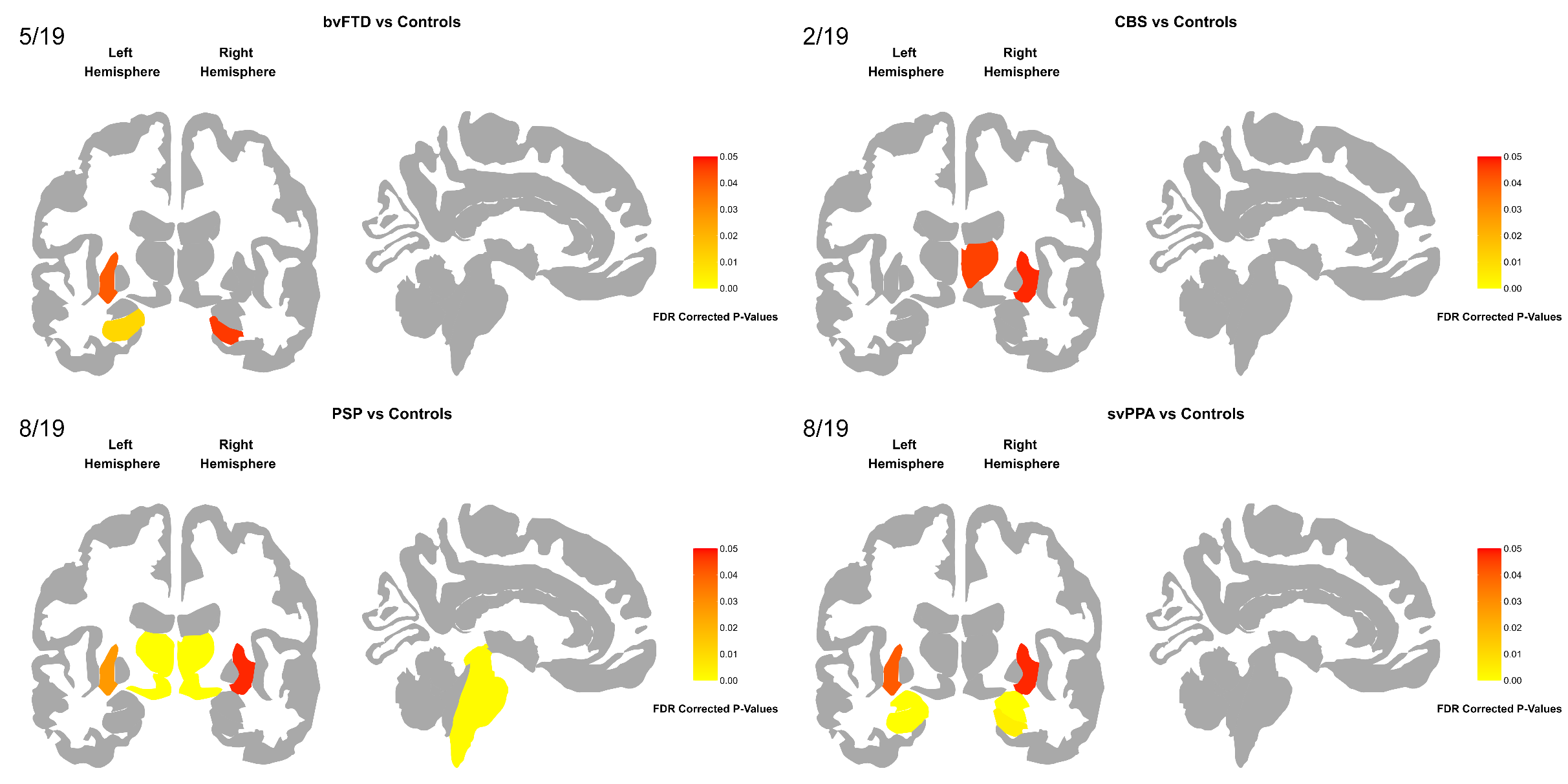


**Supplementary Figure 2B.** FDR adjusted p-value maps show significant between group differences for subcortical volumes in different diagnostic groups vs Controls at each region. **Note:** The nucleus accumbens is not depicted in the images.


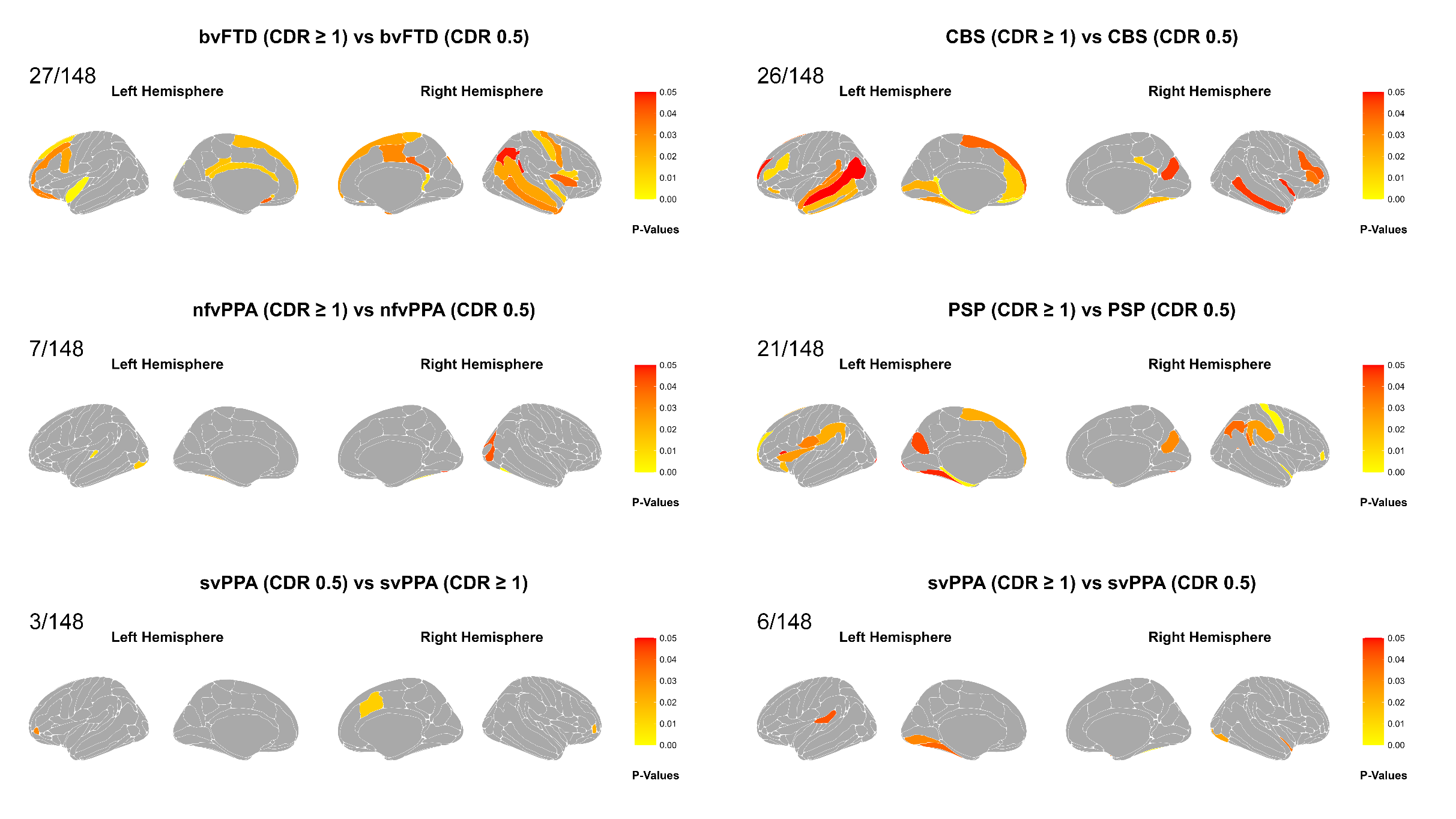


**Supplementary Figure 3A.** P-Value maps show significant within diagnostic group differences stratified by CDR/CDR^®^ plus NACC-FTLD severity for cortical thickness at each region.


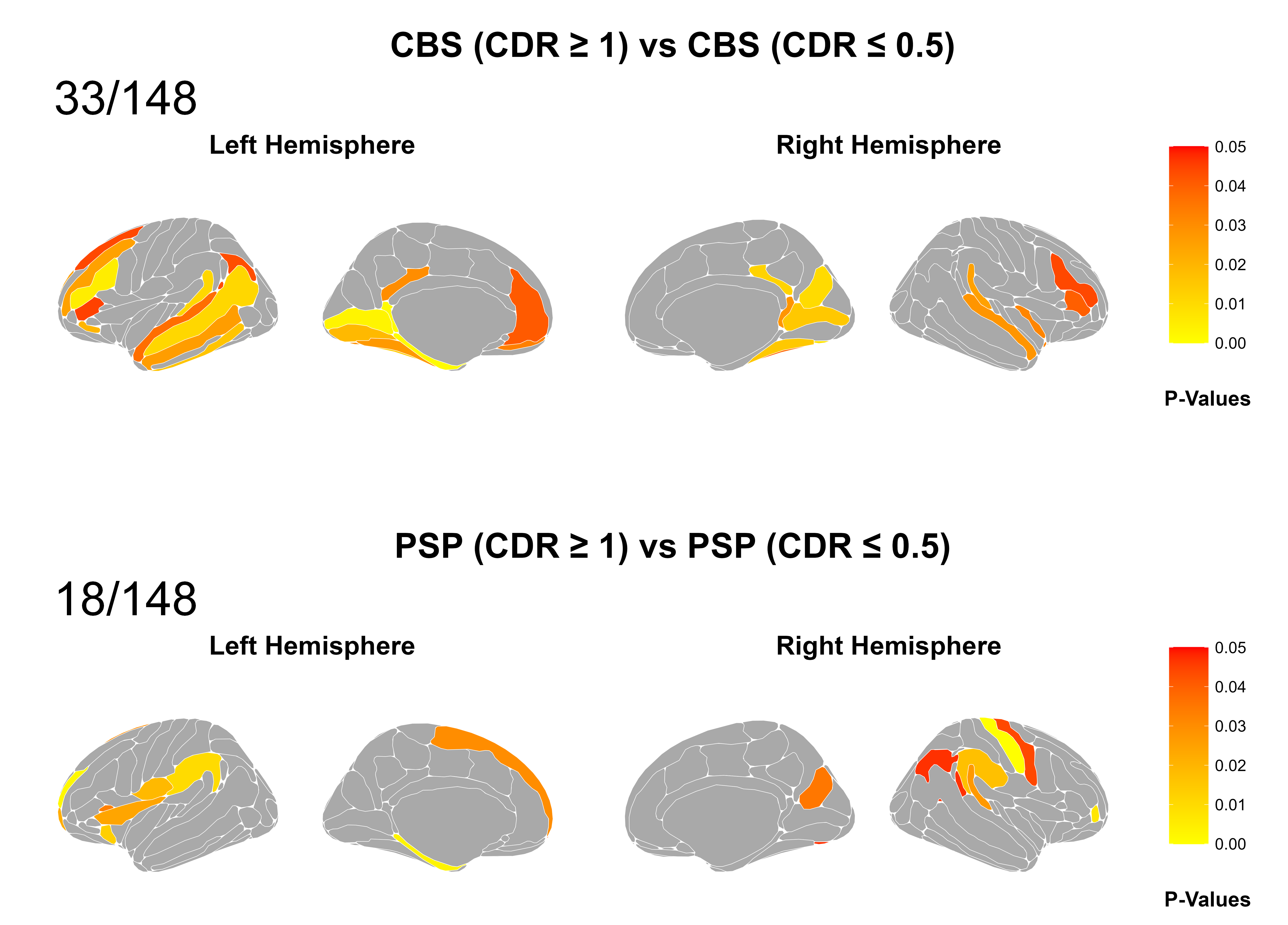


**Supplementary Figure 3A-1.** P-Value maps show significant within CBS and PSP group differences stratified by CDR severity and including individuals with CDR=0 for cortical thickness at each region.


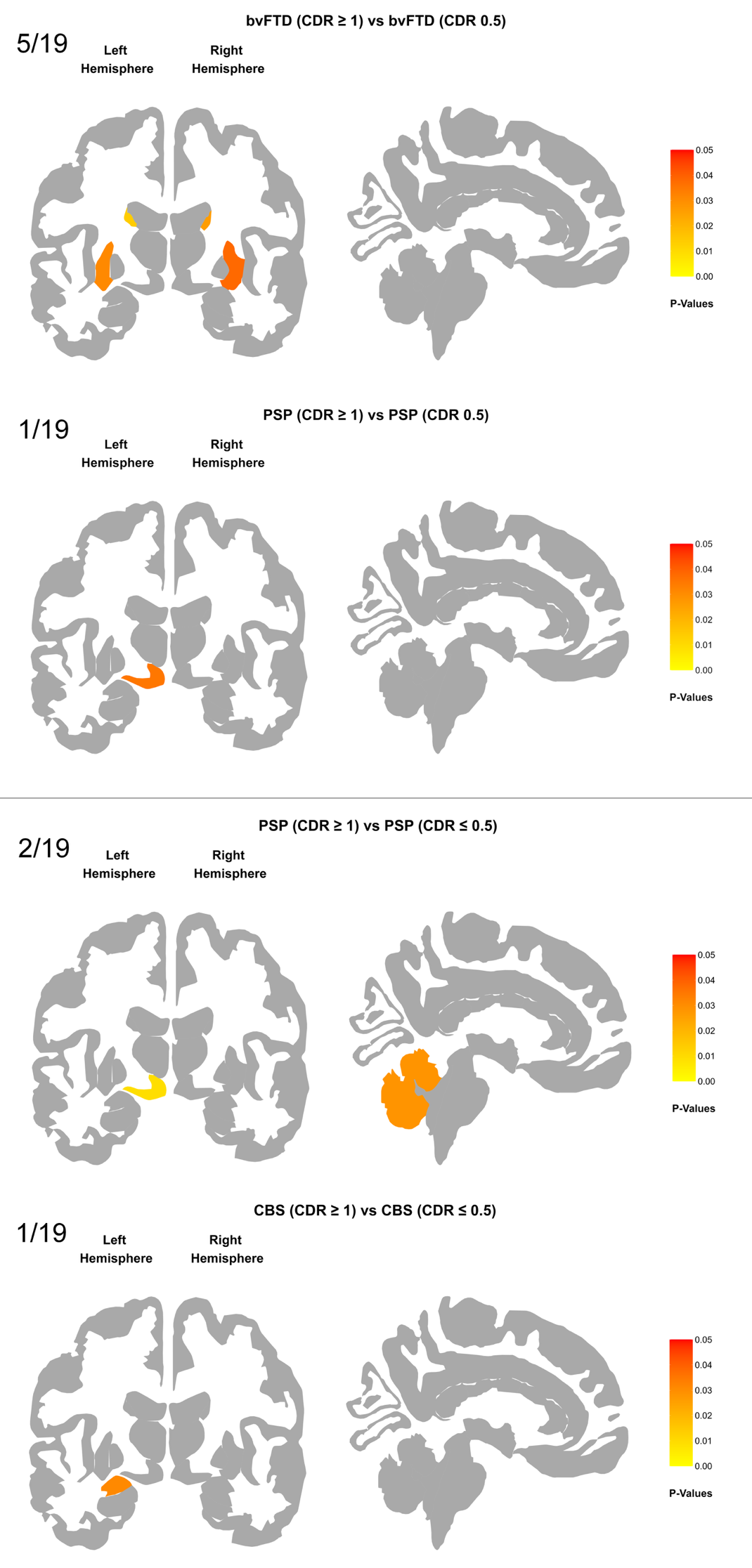


**Supplementary Figure 3B.** P-Value maps show significant within diagnostic group differences stratified by CDR/CDR^®^ plus NACC-FTLD severity for subcortical volumes at each region. **Note:** The nucleus accumbens is not depicted in the images.


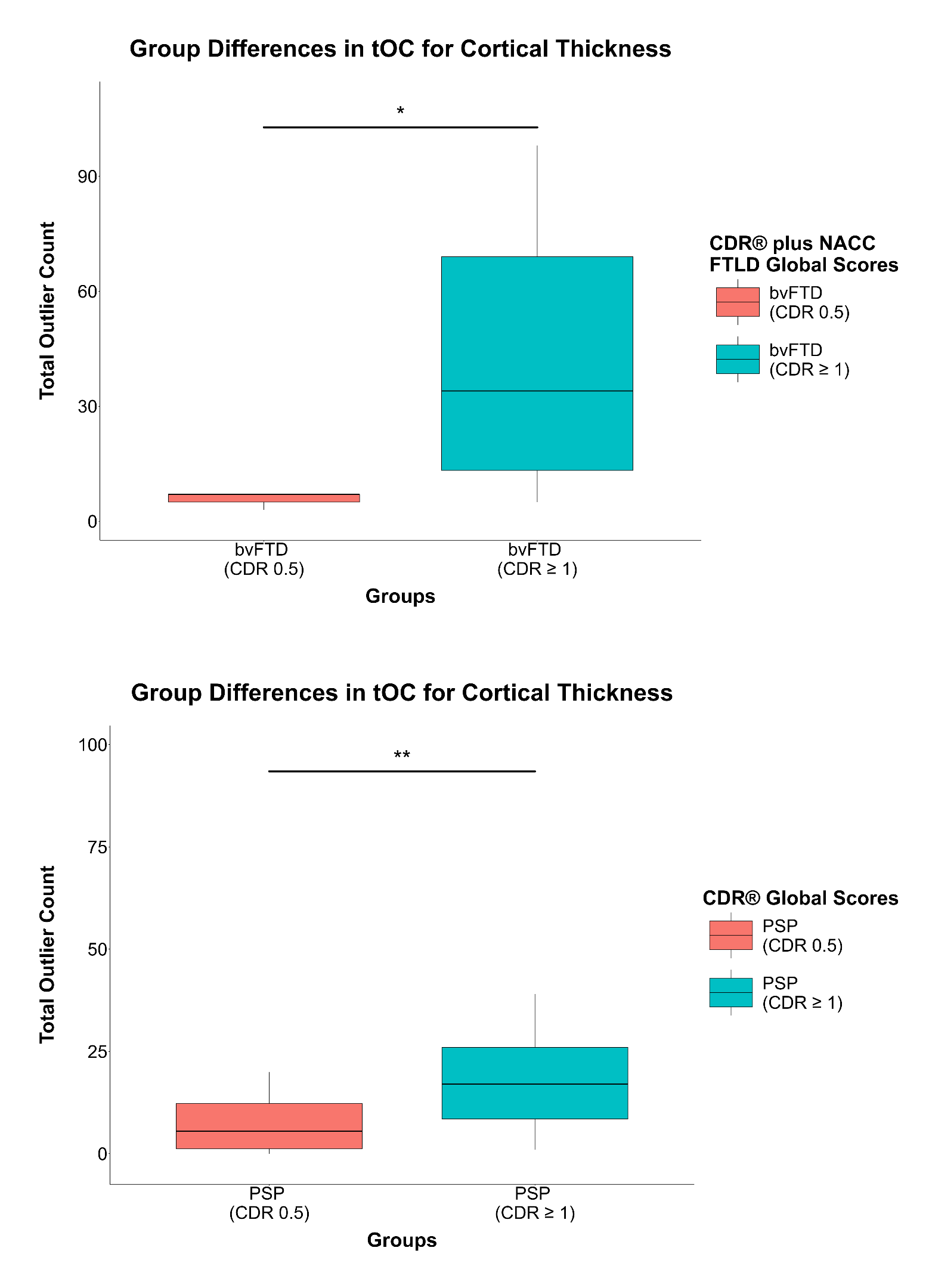


**Supplementary Figure 4A.** Pairwise comparisons using the Mann–Whitney U test shows significant between group differences in tOC for cortical thickness. The bvFTD (CDR^®^ plus NACC-FTLD ≥ 1) group has significantly higher tOC for cortical thickness than the bvFTD (CDR^®^ plus NACC-FTLD = 0.5) group and the PSP (CDR ≥ 1) group has significantly higher tOC for cortical thickness than the PSP (CDR = 0.5) group. Statistically significant p-values were denoted by the following: ^*^*p* < 0.05, ^**^*p* < 0.01.


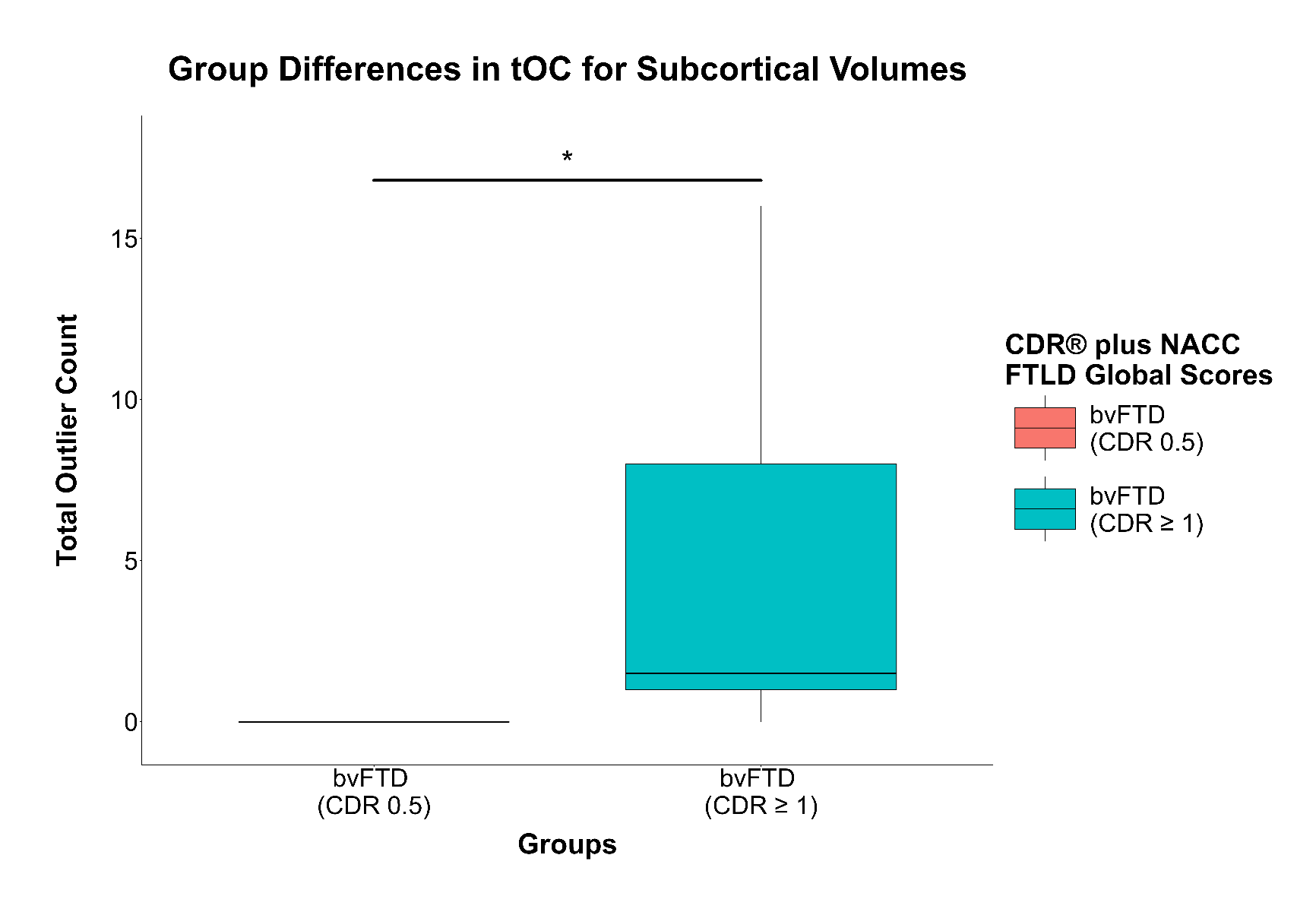


**Supplementary Figure 4B.** Pairwise comparison using the Mann–Whitney U test shows significant between group differences in tOC for subcortical volumes. The bvFTD (CDR^®^ plus NACC-FTLD ≥ 1) group has significantly higher tOC for subcortical volumes than the bvFTD (CDR^®^ plus NACC-FTLD = 0.5) group. Statistically significant p-values were denoted by the following: ^*^*p* < 0.05.


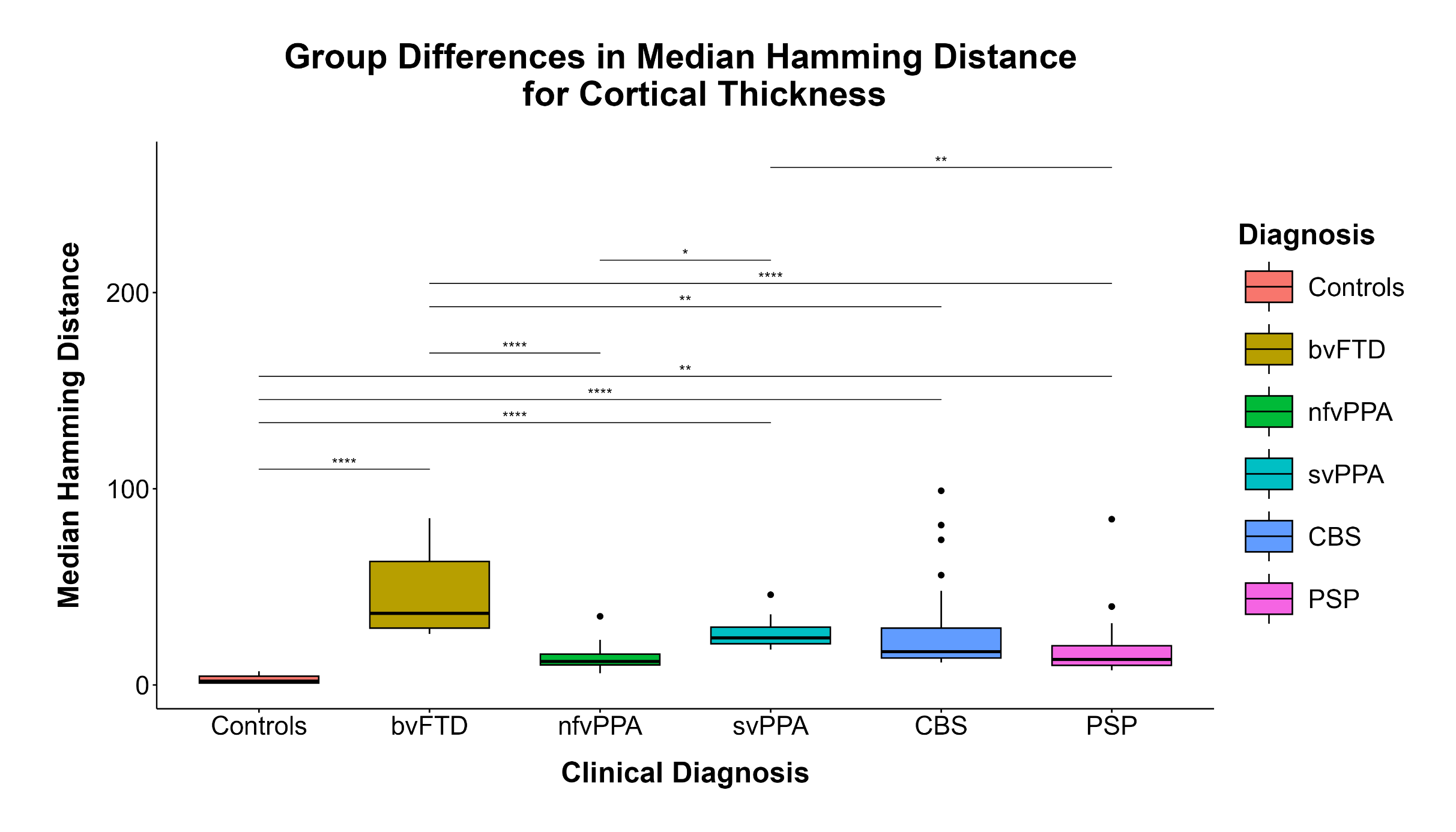


**Supplementary Figure 5A.** *Post hoc* pairwise comparisons show significant between group differences in Median Hamming Distance for cortical thickness after Bonferroni adjustment. Statistically significant p-values were denoted by the following: ^*^*p* < 0.05, ^**^*p* < 0.01, ^***^*p* < 0.001, ^****^*p* < 0.0001.


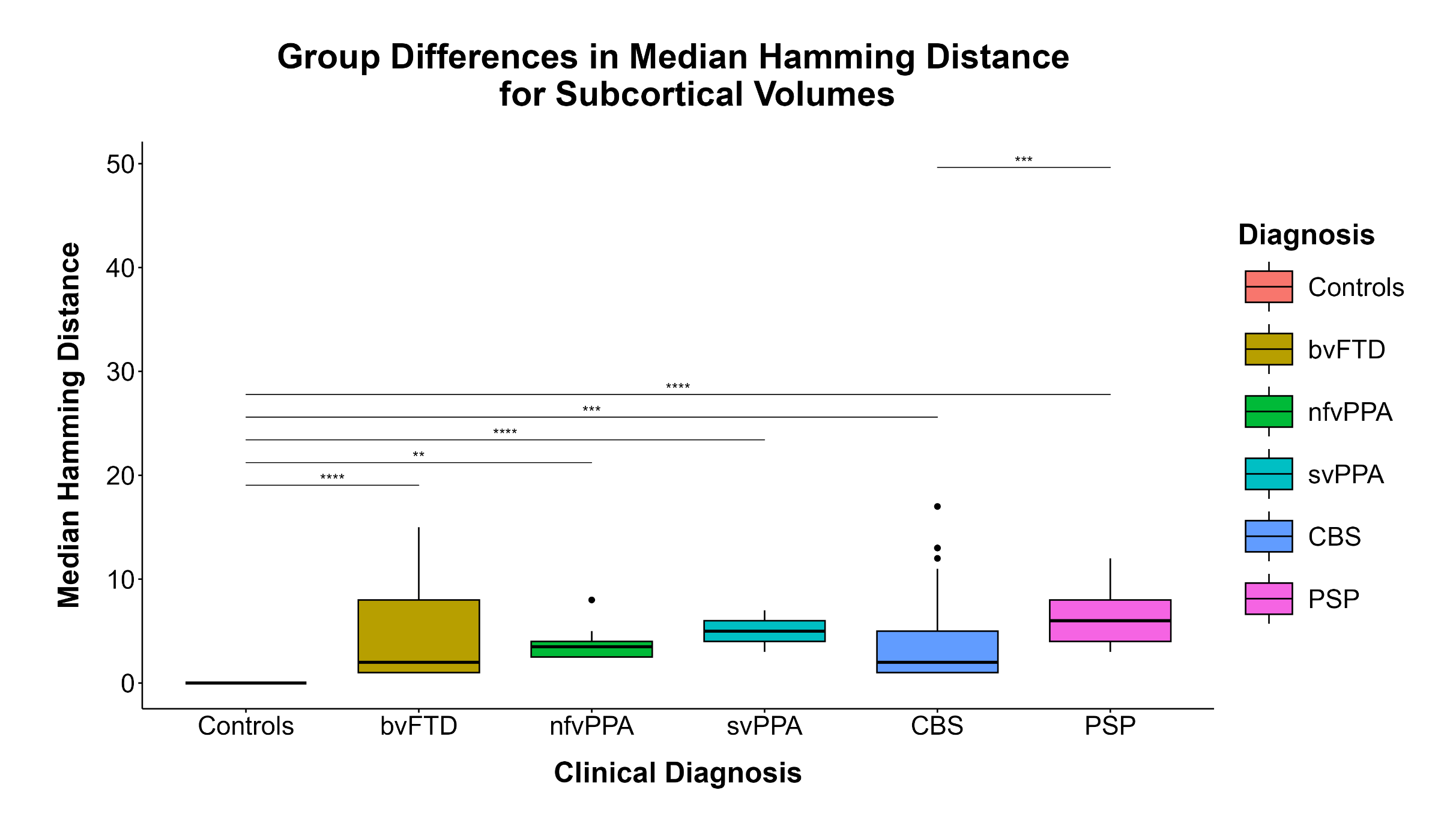


**Supplementary Figure 5B.** *Post hoc* pairwise comparisons show significant between group differences in Median Hamming Distance for subcortical volumes after Bonferroni adjustment. Statistically significant p-values were denoted by the following: ^**^*p* < 0.01, ^***^*p* < 0.001, ^****^*p* < 0.0001.


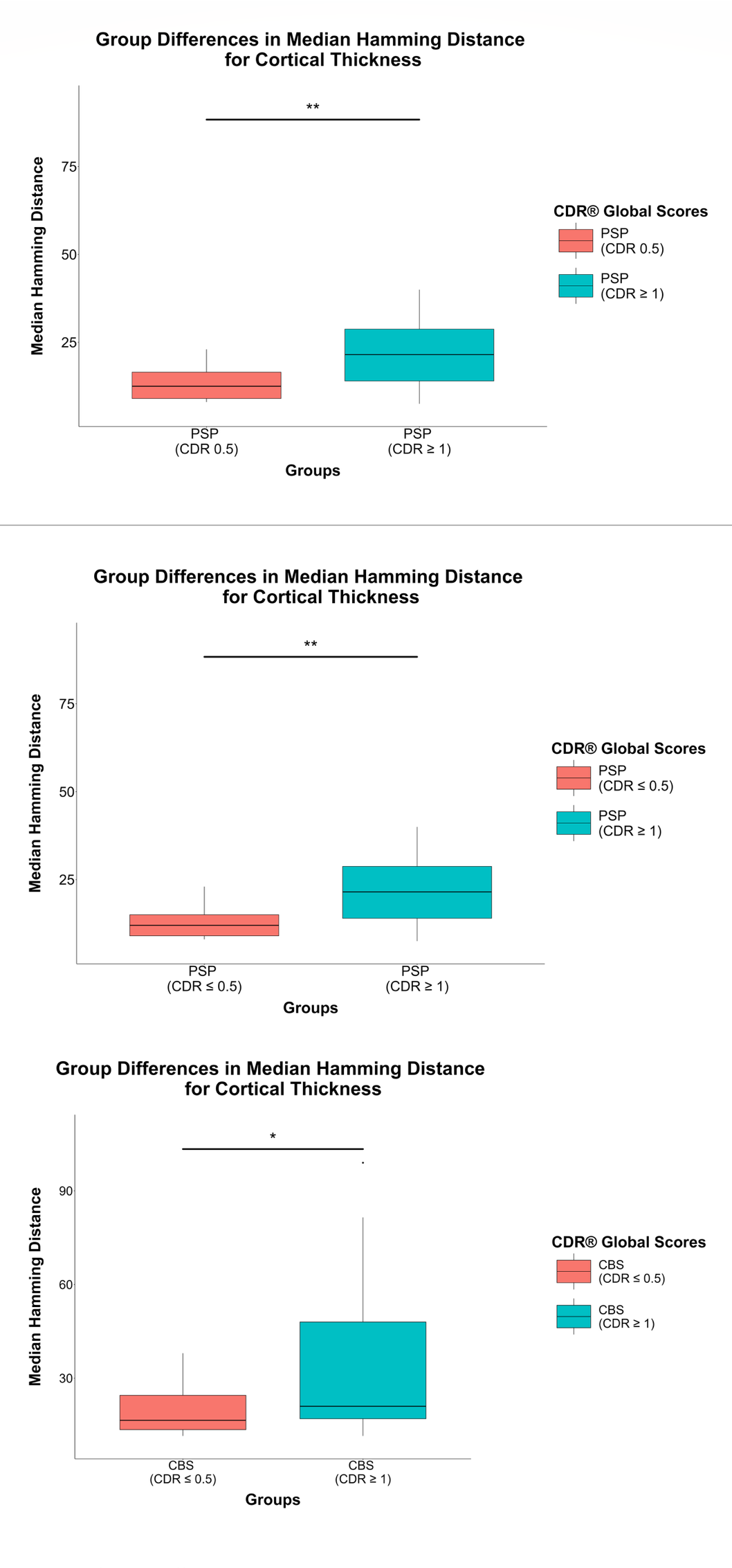


**Supplementary Figure 6A.** Pairwise comparisons using the Mann–Whitney U test shows significant between group differences in median hamming distance for cortical thickness. The PSP (CDR ≥ 1) group has significantly higher median hamming distance for cortical thickness than both PSP (CDR = 0.5) and (CDR ≤ 0.5) groups. The CBS (CDR ≥ 1) group has significantly higher median hamming distance for cortical thickness than CBS (CDR ≤ 0.5) group. Statistically significant p-values were denoted by the following: ^**^*p* < 0.01.


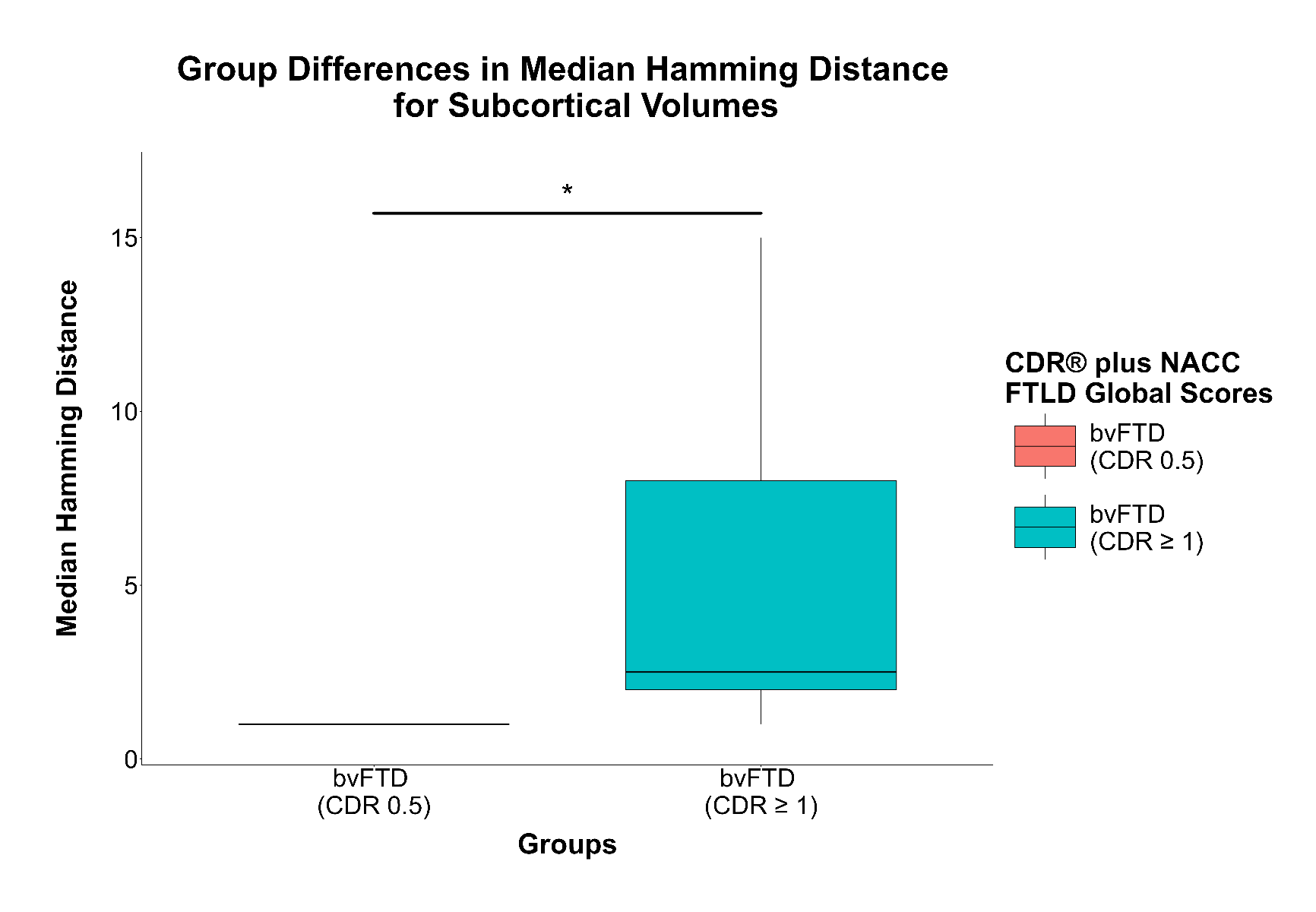


**Supplementary Figure 6B.** Pairwise comparisons using the Mann–Whitney U test shows significant between group differences in median hamming distance for subcortical volumes. The bvFTD (CDR^®^ plus NACC-FTLD ≥ 1) group has significantly higher median hamming distance for subcortical volumes than the bvFTD (CDR^®^ plus NACC-FTLD = 0.5) group. Statistically significant p-values were denoted by the following: ^*^*p* < 0.05.


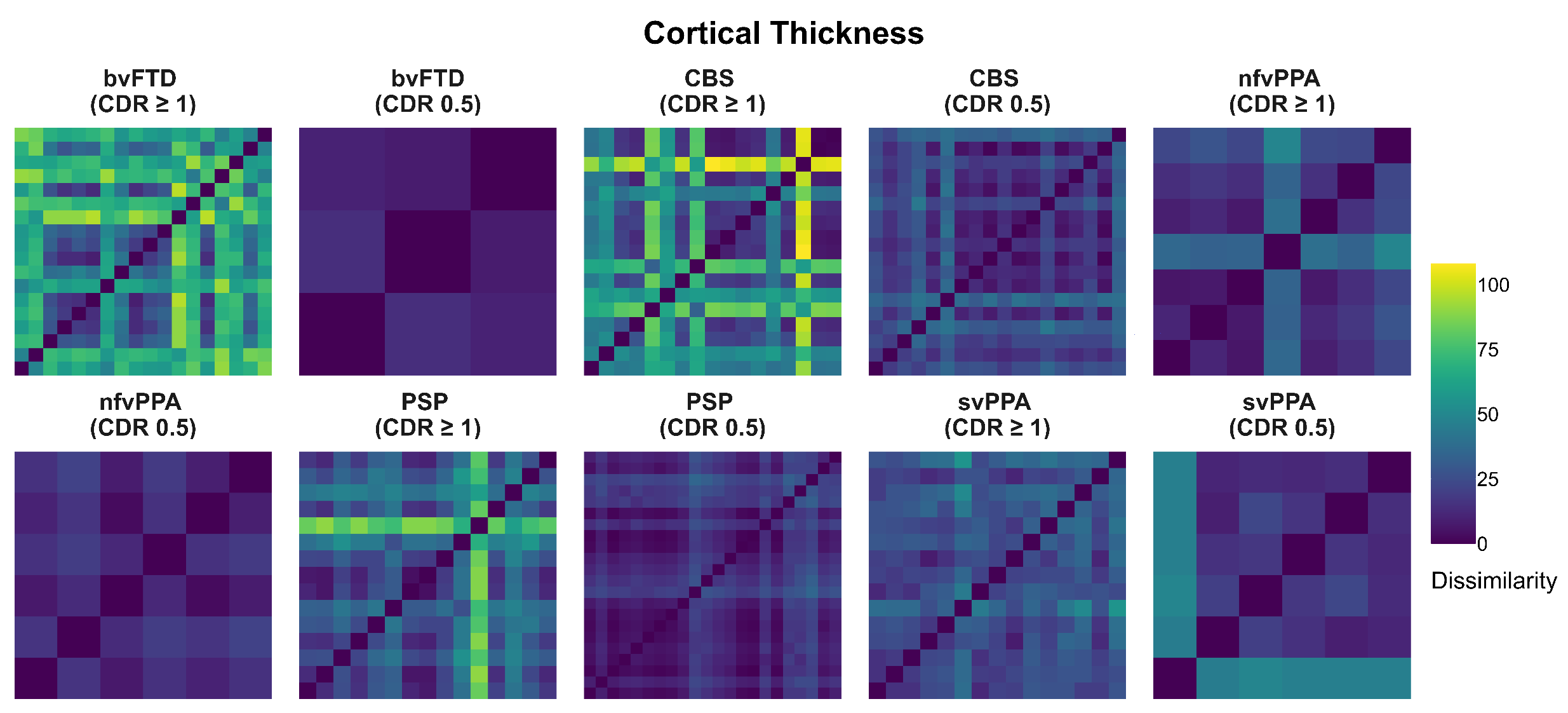


**Supplementary Figure 7A.** Hamming distance heatmaps for cortical thickness within each diagnostic group stratified by CDR/CDR^®^ plus NACC-FTLD severity (CDR ≥ 1 vs. CDR = 0.5). In general, the CDR = 0.5 groups have very low Hamming distance values as depicted by the dark blue/light blue/dark green colours indicating very low dissimilarity than the CDR ≥ 1 groups who have very high Hamming distance values as depicted by green/yellow colours.


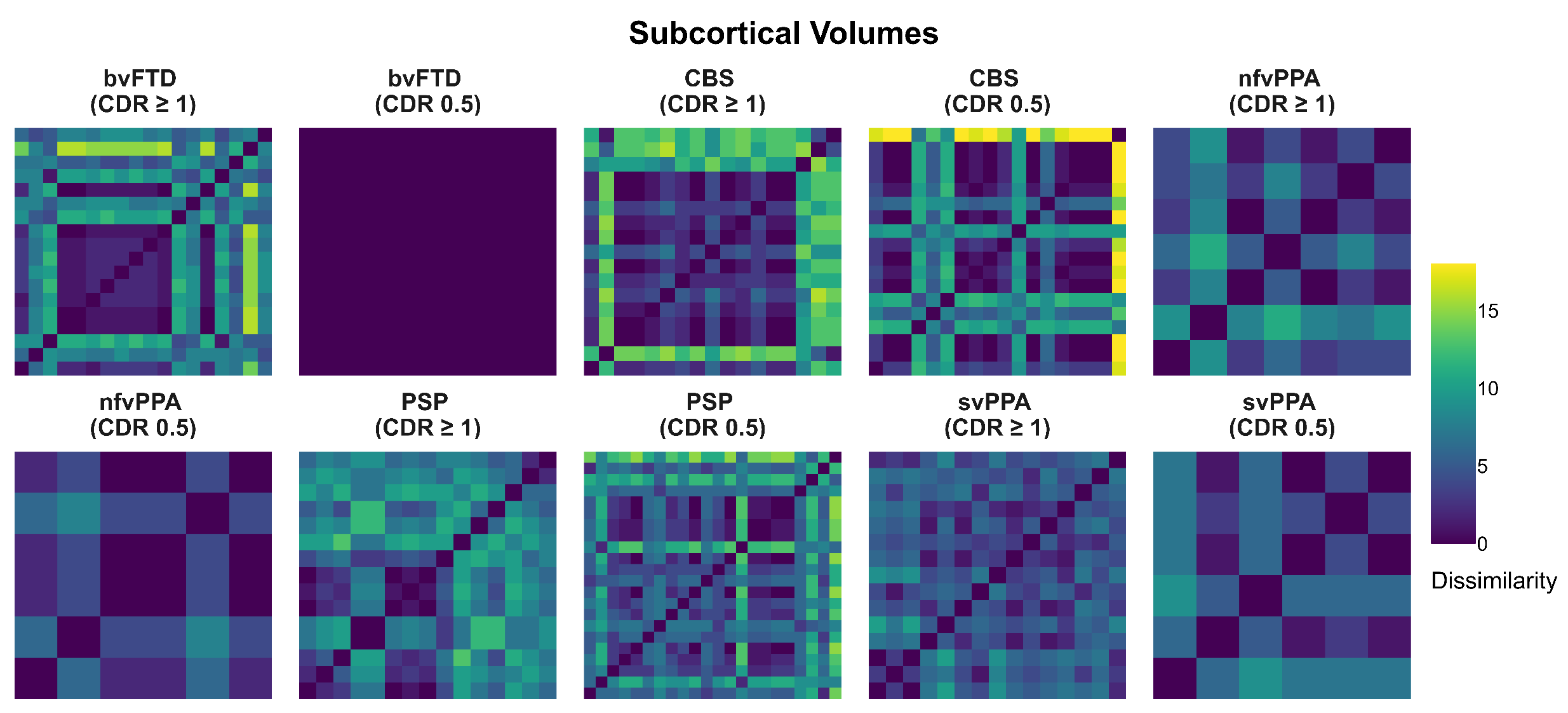


**Supplementary Figure 7B.** Hamming distance heatmaps for subcortical volumes within each diagnostic group stratified by CDR/CDR^®^ plus NACC-FTLD severity (CDR ≥ 1 vs. CDR = 0.5). Unlike cortical thickness, most of the CDR = 0.5 groups (except bvFTD) have slightly higher Hamming distance values as depicted by the dark blue/light green-yellow colours indicating almost comparable but marginally lower dissimilarity than the CDR ≥ 1 groups who have very high Hamming distance values as depicted by green/yellow colours.


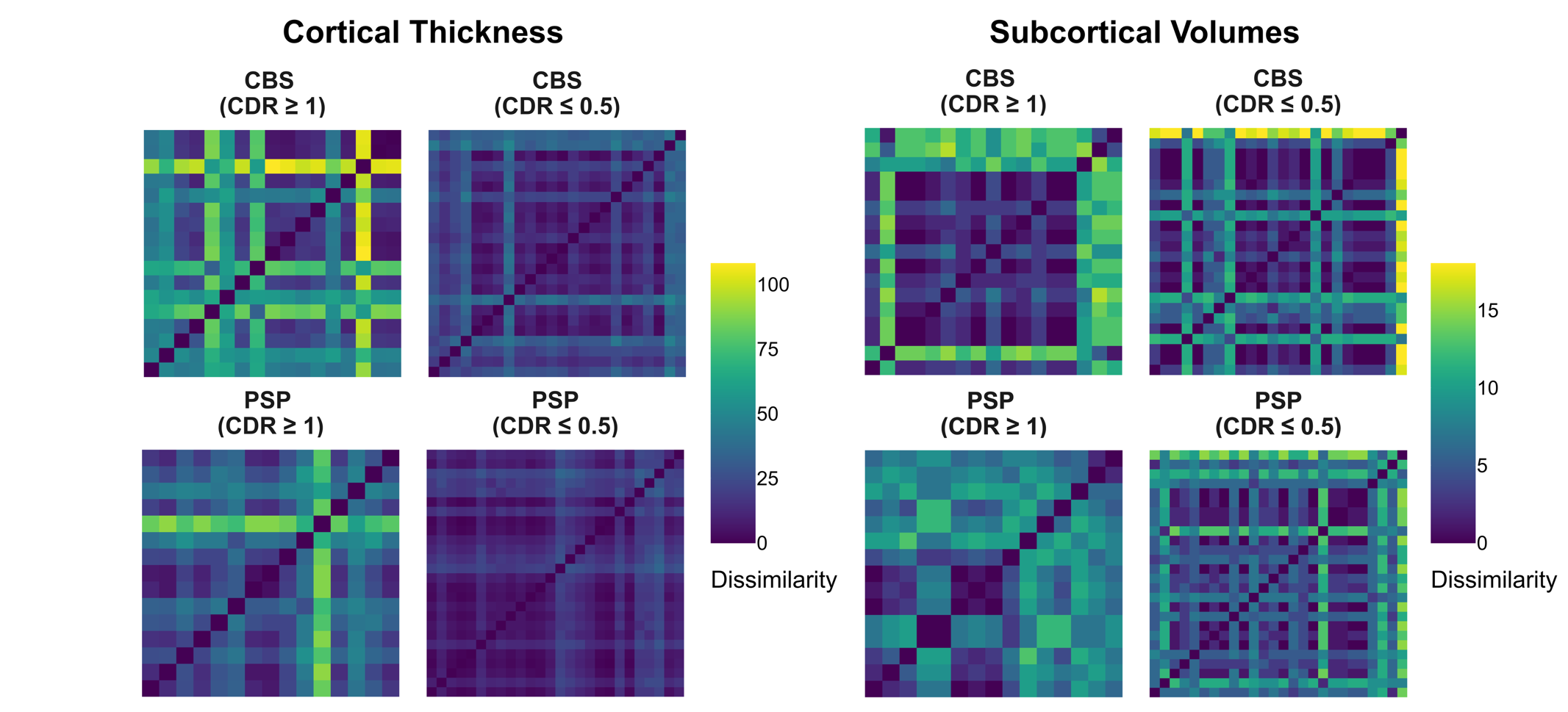


**Supplementary Figure 7C.** Hamming distance heatmaps for cortical thickness and subcortical volumes within CBS and PSP groups stratified by CDR global scores (CDR ≥ 1 vs. CDR ≤ 0.5). For cortical regions, CDR ≥ 1 groups have higher Hamming distance values than CDR ≤ 0.5 groups as depicted by green/yellow colours.


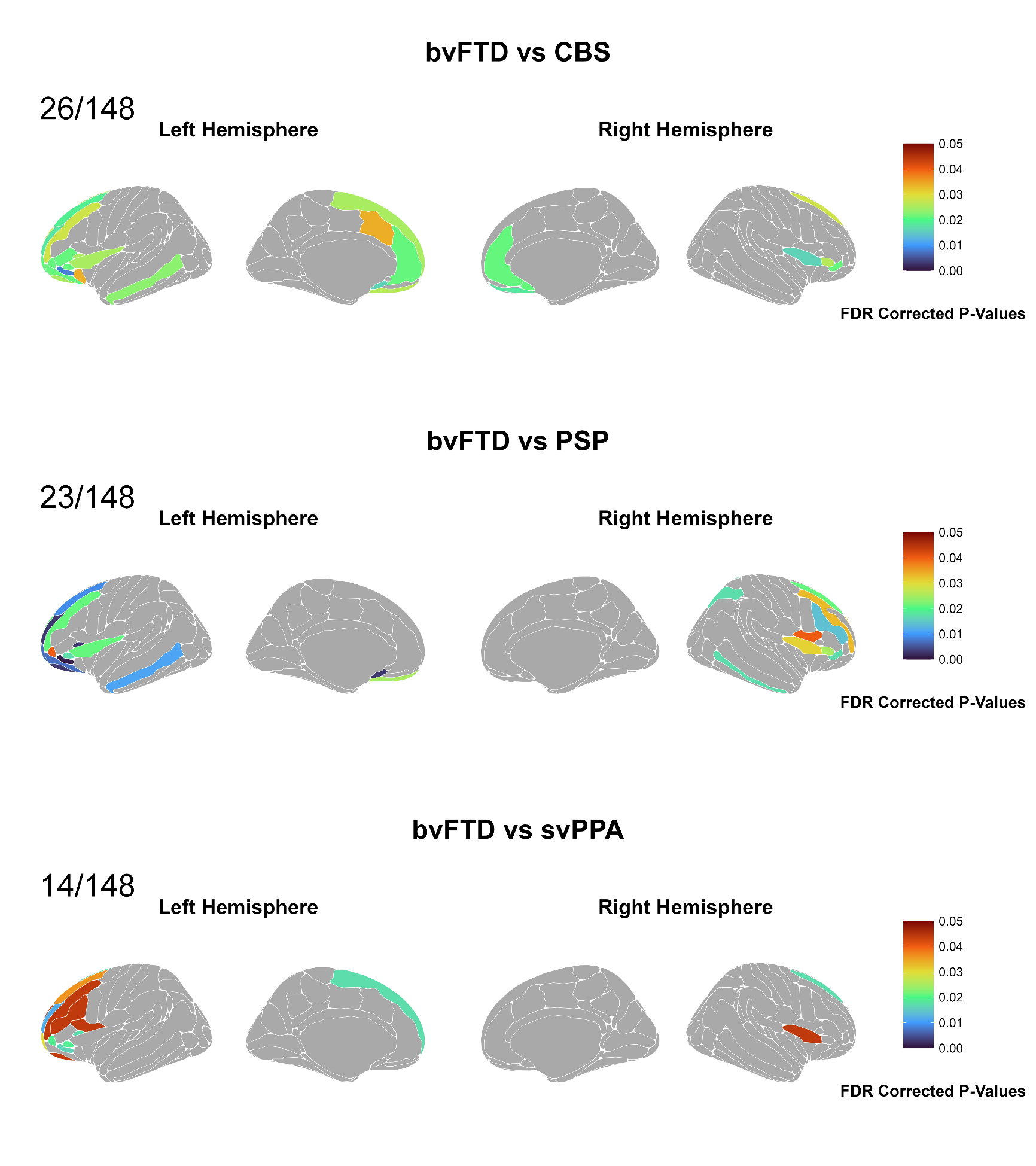


**Supplementary Figure 8A.** FDR adjusted p-value maps show significant between group differences for cortical thickness outliers in bvFTD at each region.


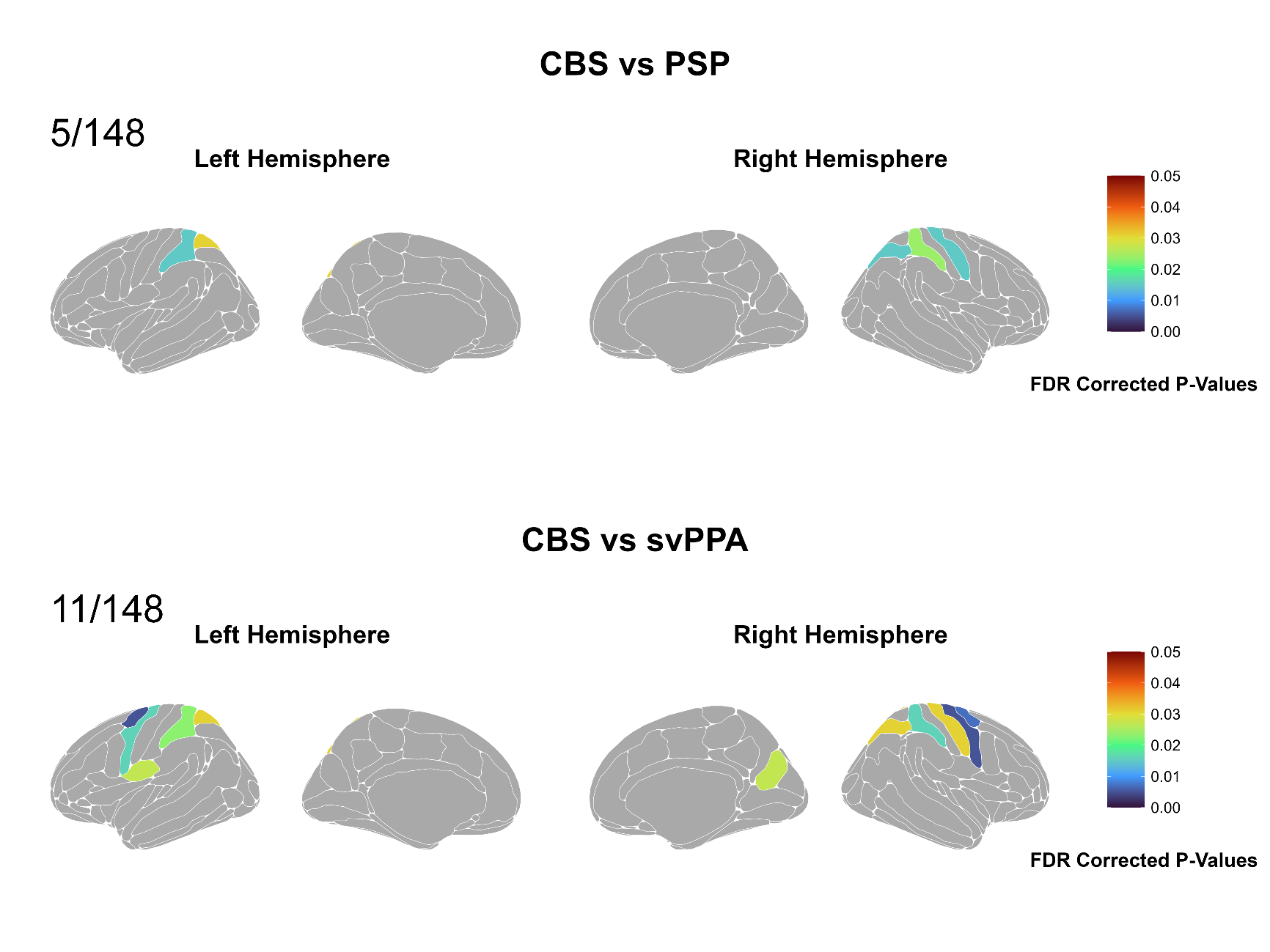


**Supplementary Figure 8B.** FDR adjusted p-value maps show significant between group differences for cortical thickness outliers in CBS at each region.


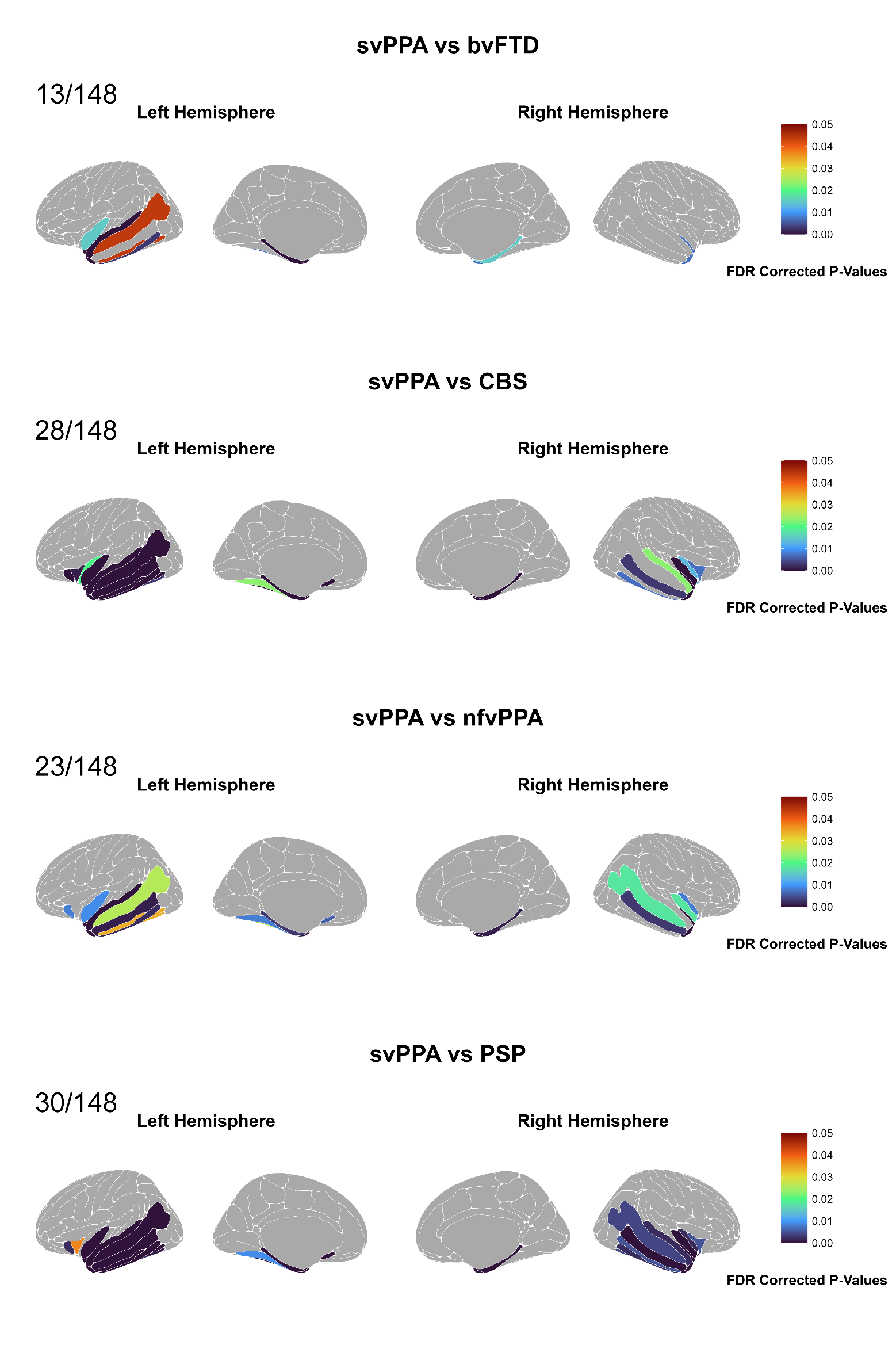


**Supplementary Figure 8C.** FDR adjusted p-value maps show significant between group differences for cortical thickness outliers in svPPA at each region.


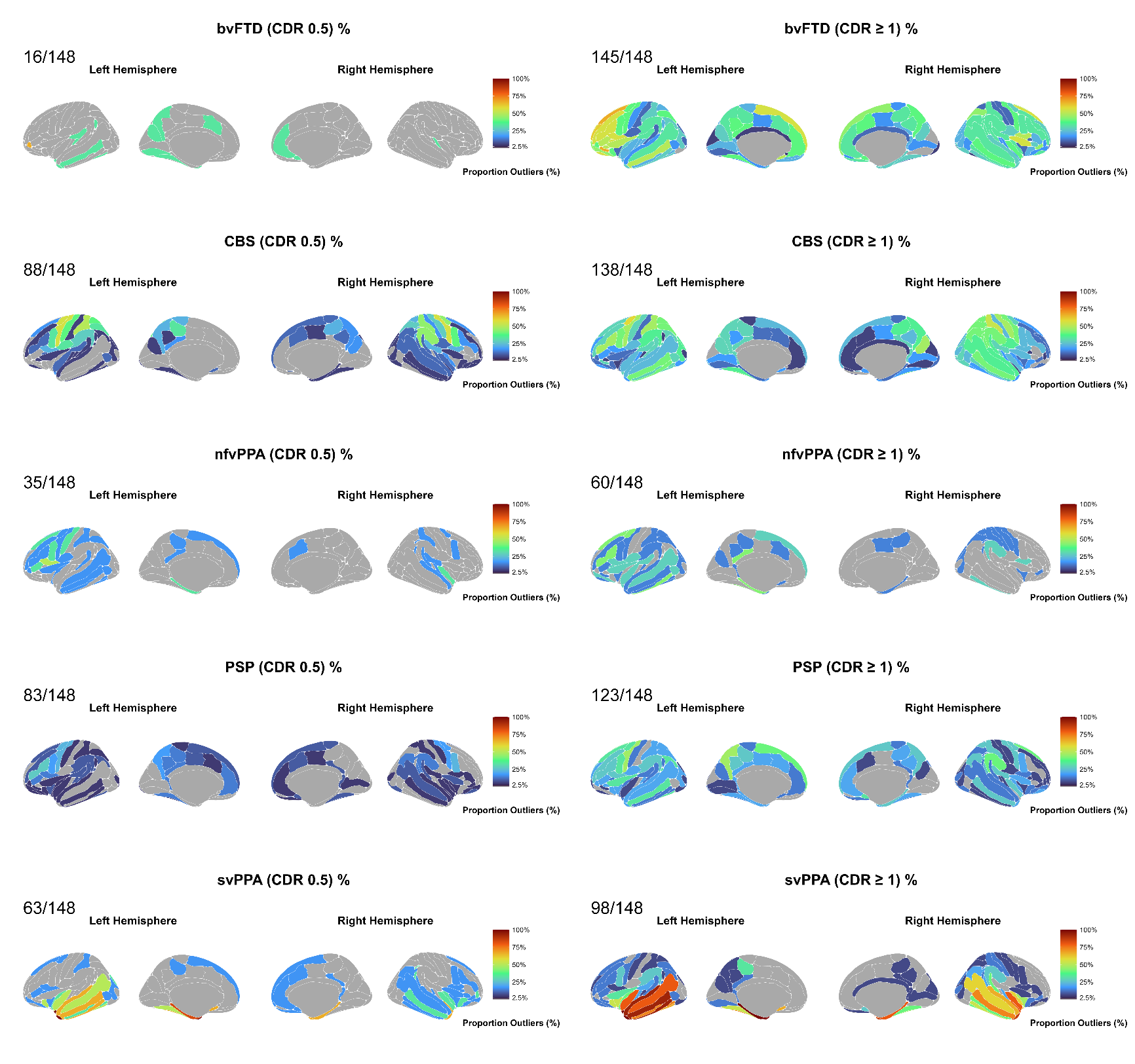


**Supplementary Figure 9A.** Percentage of patients with outliers stratified by CDR/CDR^®^ plus NACC-FTLD severity (CDR ≥ 1 vs. CDR = 0.5) that have been mapped at each of the 148 regions. The colour bar reflects the percentage of outliers from 0% (darker colours such as dark blue) to 100% (bright colours such as dark orange). The grey colour represents regions where the proportion of outliers is between 0 and < 2.5%.


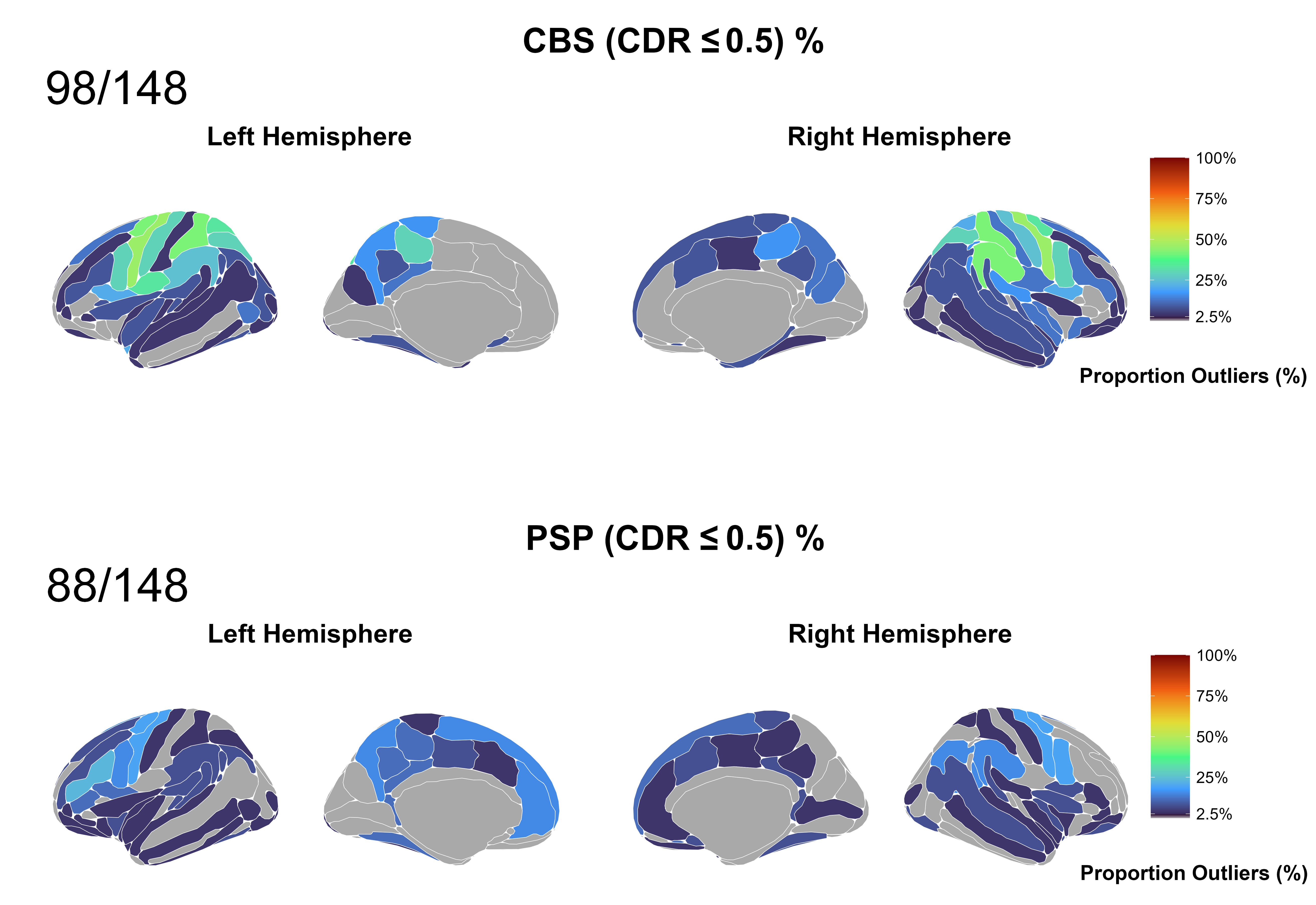


**Supplementary Figure 9B.** Percentage CBS and PSP patients with CDR global scores ≤ 0.5 that have been mapped at each of the 148 regions. The colour bar reflects the percentage of outliers from 0% (darker colours such as dark blue) to 100% (bright colours such as dark orange). The grey colour represents regions where the proportion of outliers is between 0 and < 2.5%.


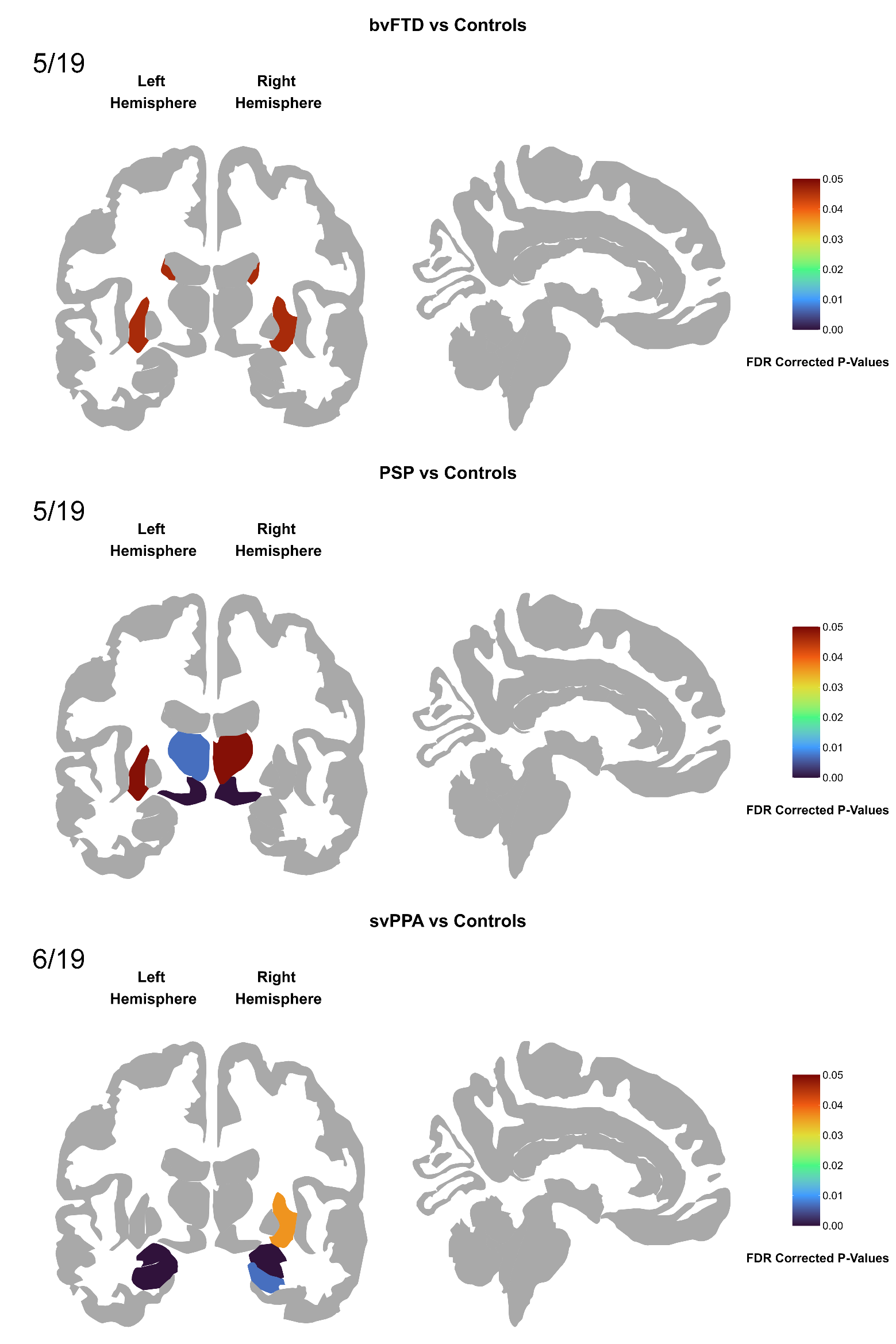


**Supplementary Figure 10.** FDR adjusted p-value maps show significant between group differences for subcortical volume outliers in different diagnostic groups vs Controls at each region. **Note:** The nucleus accumbens is not depicted in the images.


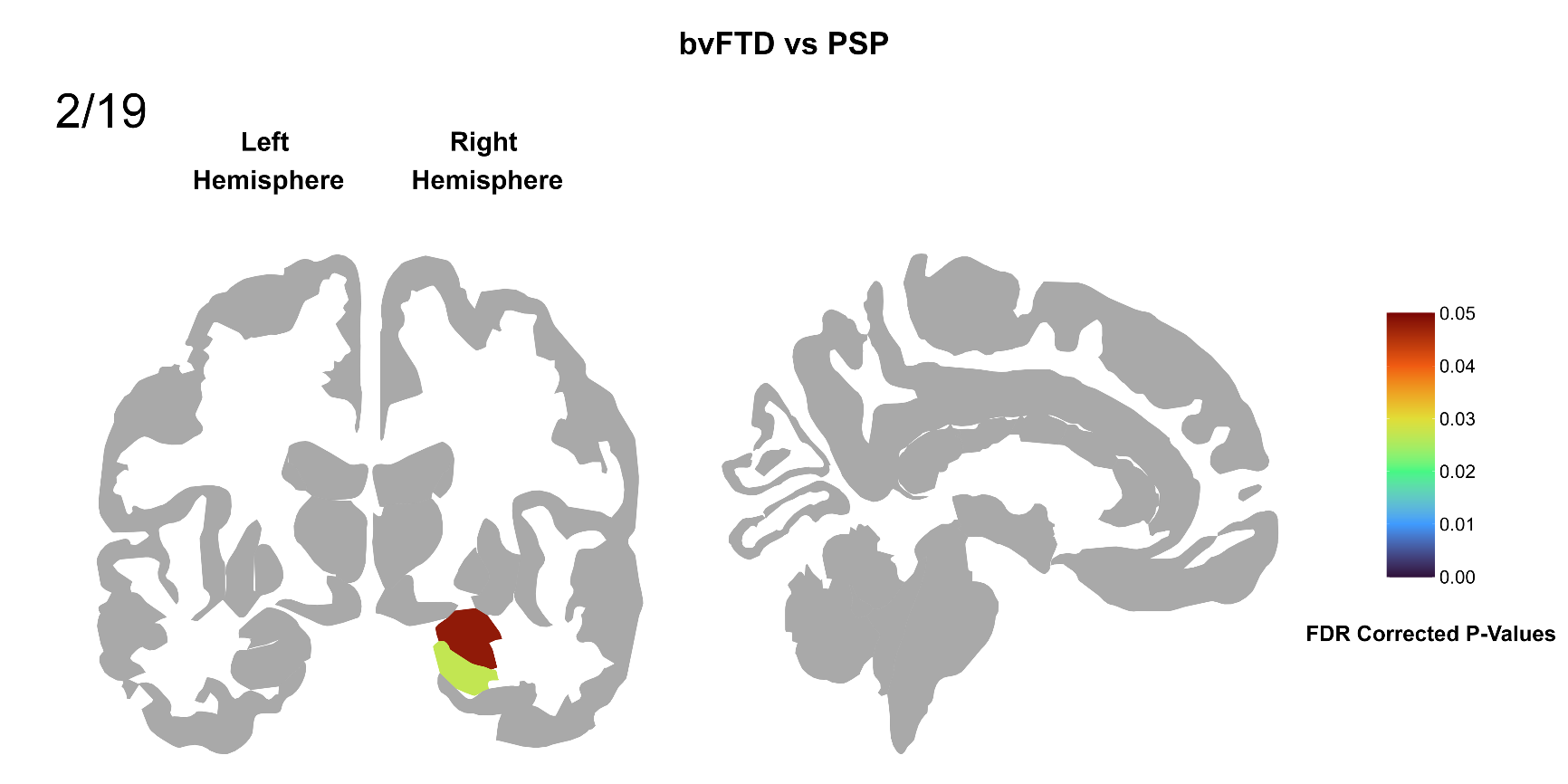


**Supplementary Figure 11A.** FDR adjusted p-value maps show significant between group differences for subcortical volume outliers in bvFTD vs diagnostic group at each region.


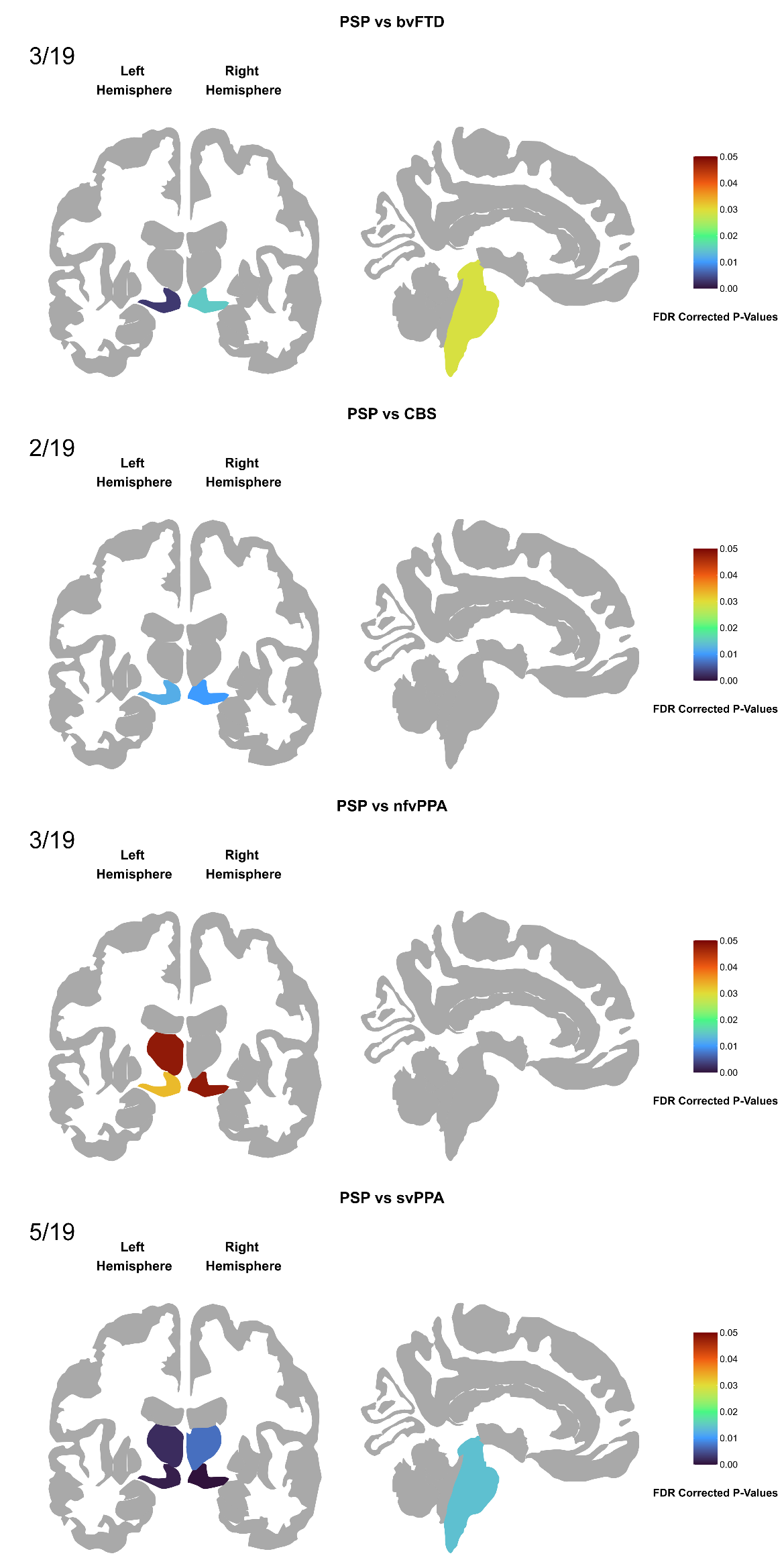


**Supplementary Figure 11B.** FDR adjusted p-value maps show significant between group differences for subcortical volume outliers in PSP vs diagnostic group at each region.


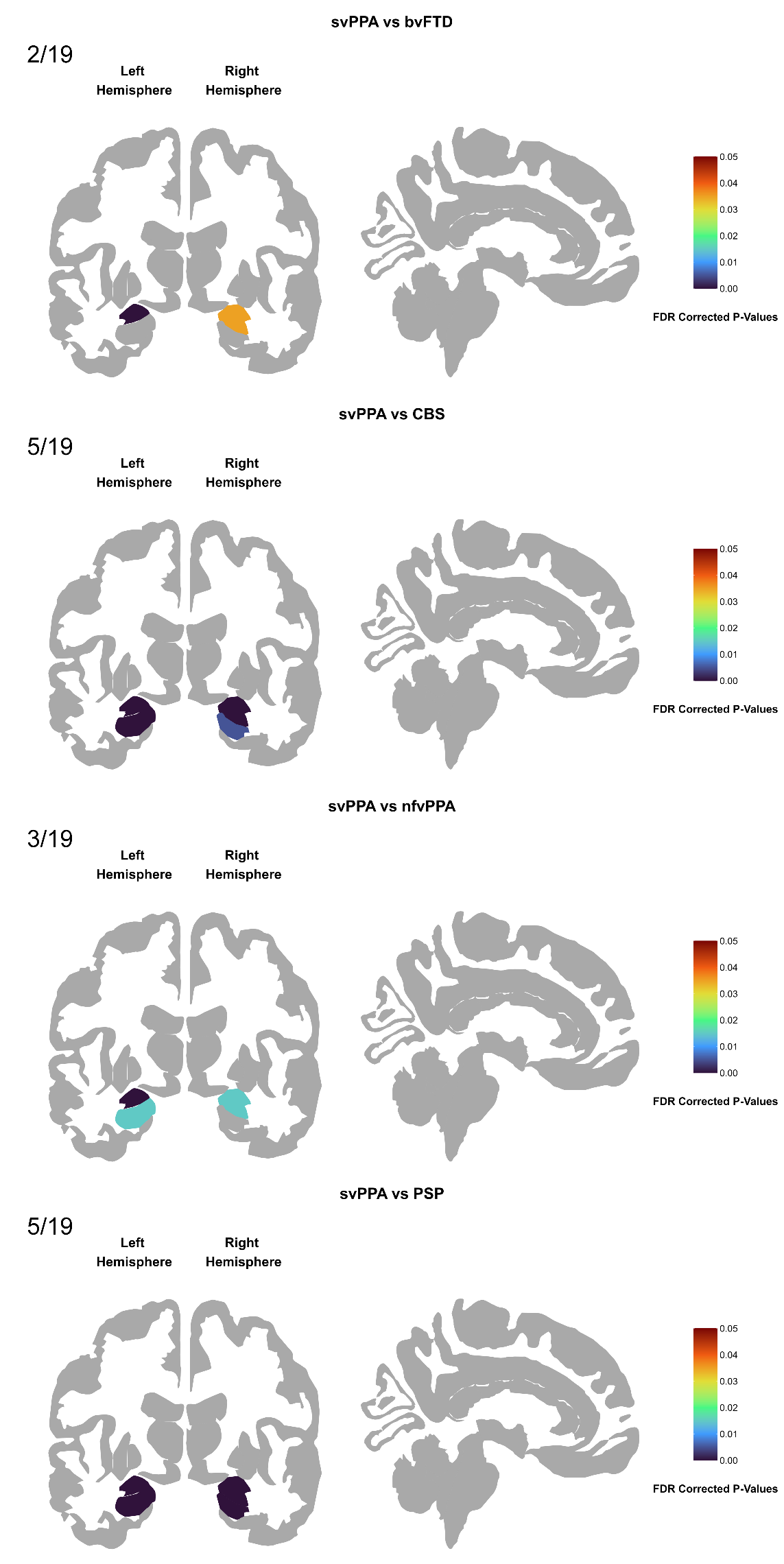


**Supplementary Figure 11C.** FDR adjusted p-value maps show significant between group differences for subcortical volume outliers in svPPA vs diagnostic group at each region. **Note:** The nucleus accumbens is not depicted in any of the images.


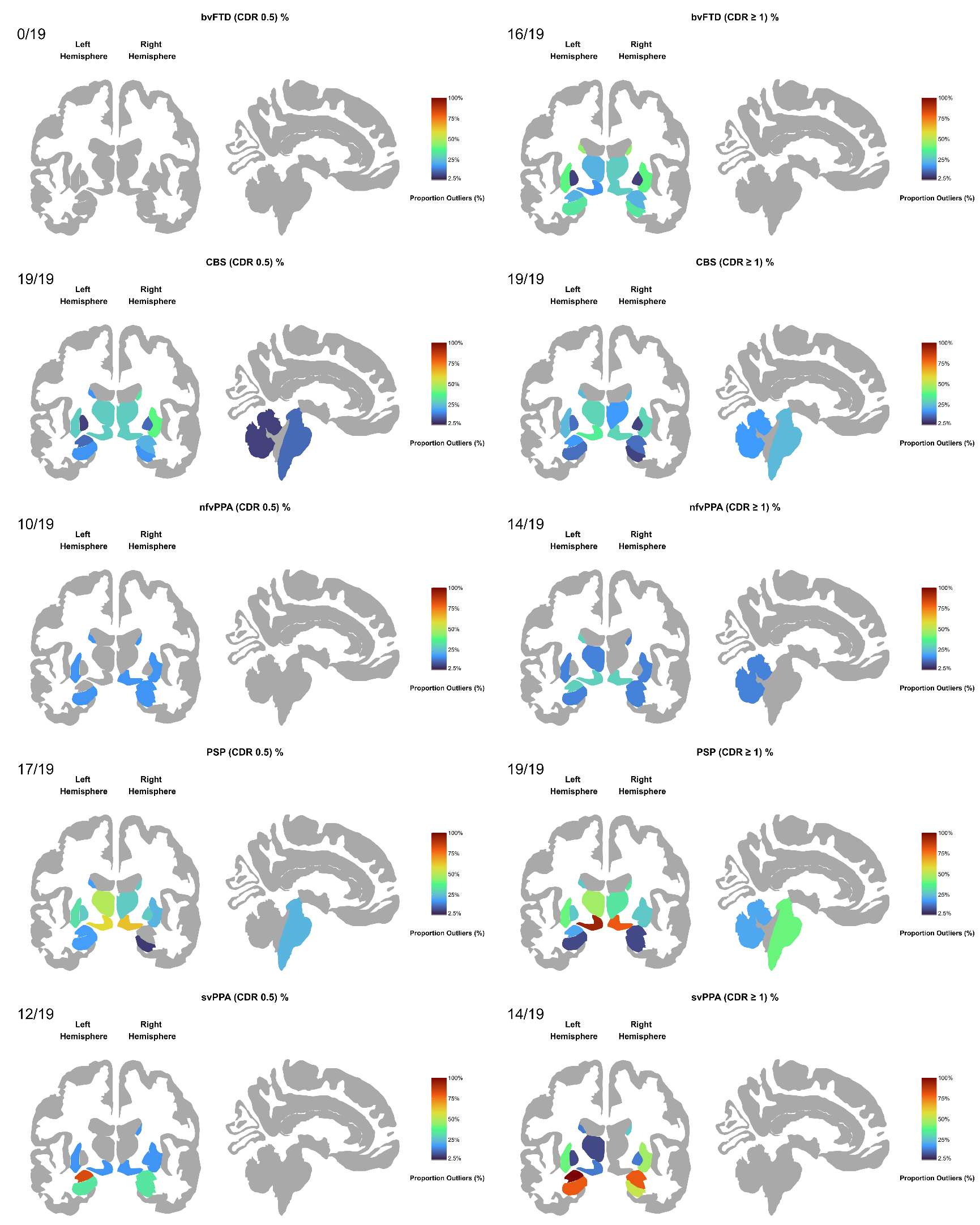


**Supplementary Figure 12A.** Percentage of patients with outliers stratified by CDR/CDR^®^ plus NACC-FTLD severity (CDR ≥ 1 vs. CDR = 0.5) that have been mapped at each of the 19 regions. The colour bar reflects the percentage of outliers from 0% (darker colours such as dark blue) to 100% (bright colours such as dark orange). The grey colour represents regions where the proportion of outliers is between 0 and < 2.5%. **Note:** The nucleus accumbens is not depicted in the images.

**Supplementary Figure 12.** Percentage of patients with outliers stratified by CDR/CDR^®^ plus NACC-FTLD severity (CDR ≥ 1 vs. CDR = 0.5) that have been mapped at each of the 19 regions. The colour bar reflects the percentage of outliers from 0% (darker colours such as dark blue) to 100% (bright colours such as dark orange). The grey colour represents regions where the proportion of outliers is between 0 and < 2.5%. **Note:** The nucleus accumbens is not depicted in the images.


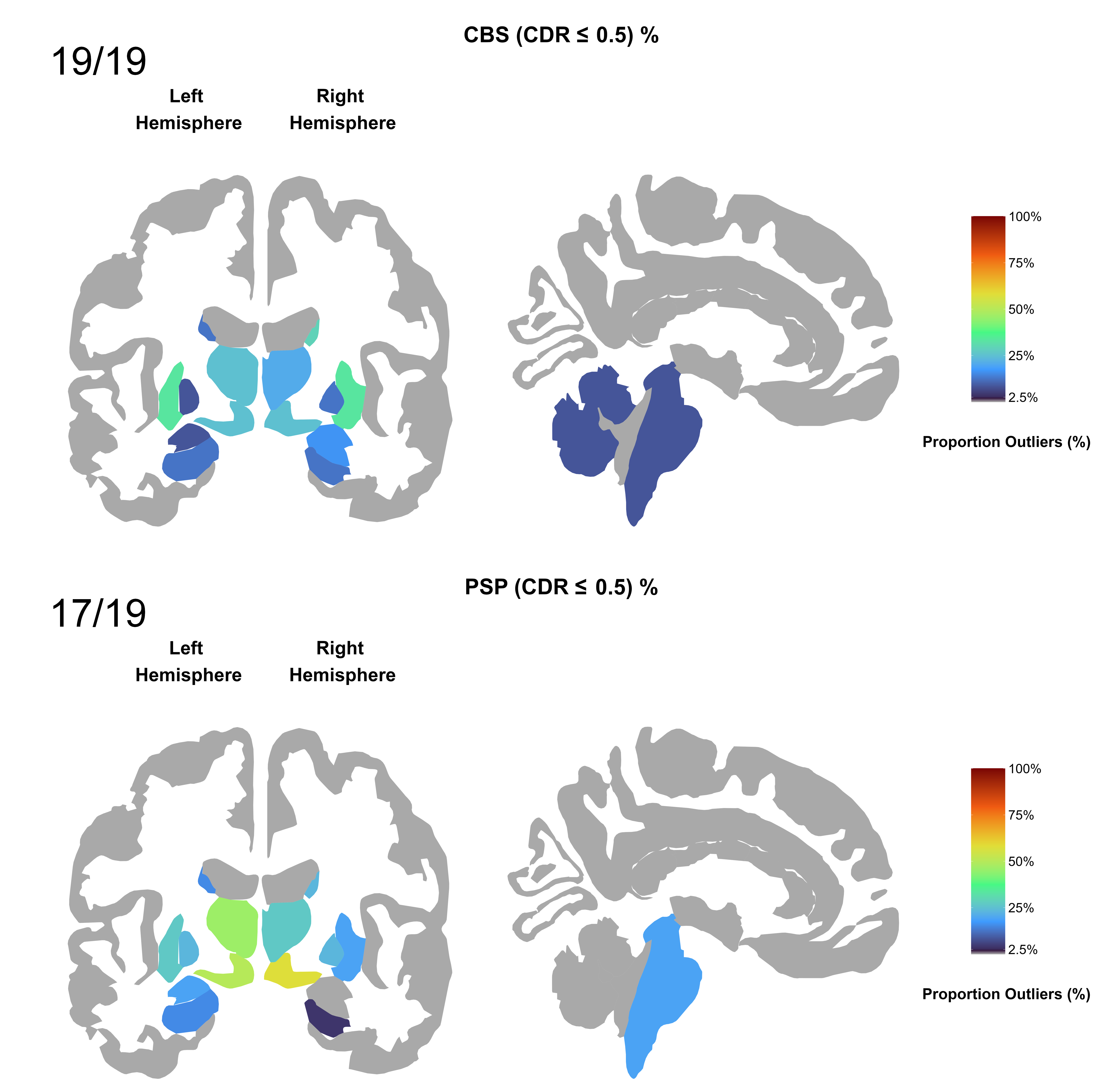


**Supplementary Figure 12B.** Percentage of CBS and PSP patients with CDR global scores ≤ 0.5 that have been mapped at each of the 19 regions. The colour bar reflects the percentage of outliers from 0% (darker colours such as dark blue) to 100% (bright colours such as dark orange). The grey colour represents regions where the proportion of outliers is between 0 and < 2.5%. **Note:** The nucleus accumbens is not depicted in the images.

**Supplementary Table 1**. Number of patients for each severity subgroups included in the analyses. From the original experimental sample of patients who passed quality control (N = 145), a total of 8 patients were excluded due to missing data: 2 with CBS, 4 with PSP, 1 with bvFTD, and 1 with nfvPPA.

|  | **CDR global score** | | | **CDR^®^ plus NACC-FTLD global score** | | |
| --- | --- | --- | --- | --- | --- | --- |
|  | **0** | **0.5** | **1 or over** | **0** | **0.5** | **1 or over** |
| **CBS**  **(N=41)** | 6 | 18 | 17 | -- | -- | -- |
| **PSP**  **(N=41)** | 4 | 22 | 15 | -- | -- | -- |
| **bvFTD**  **(N=21)** | 0 | 6 | 15 | 0 | 3 | 18 |
| **nfvPPA**  **(N=13)** | 2 | 11 | 0 | 0 | 6 | 7 |
| **svPPA**  **(N=21)** | 0 | 16 | 5 | 0 | 6 | 15 |
